# Synthesis and Biological Activity Assessment of 2-Styrylbenzothiazoles as Potential Multifunctional Therapeutic Agents

**DOI:** 10.3390/antiox13101196

**Published:** 2024-10-01

**Authors:** Riccardo Barbari, Vera Bruggink, Robert Klaus Hofstetter, Chiara Tupini, Sofia Fagnani, Erika Baldini, Elisa Durini, Ilaria Lampronti, Silvia Vertuani, Anna Baldisserotto, Oliver Werz, Stefano Manfredini

**Affiliations:** 1 Department of Life Science and Biotechnology, Section of Medicines and Health Products, University of Ferrara, Via Fossato di Mortara 17-19, I-44121 Ferrara, Italy; brbrcr@unife.it (R.B.); bldrke@unife.it (E.B.); dre@unife.it (E.D.); vrs@unife.it (S.V.); smanfred@unife.it (S.M.); 2 Department of Pharmaceutical/Medicinal Chemistry, Institute of Pharmacy, Friedrich Schiller University Jena, 07743 Jena, Germany; vera.bruggink@uni-jena.de (V.B.); robert.klaus.hofstetter@uni-jena.de (R.K.H.); 3 Department of Life Science and Biotechnology, Section of Biochemistry and Molecular Biology, University of Ferrara, Via Fossato di Mortara 74, I-44121 Ferrara, Italy; chiara.tupini@unife.it (C.T.); sofia.fagnani@unife.it (S.F.); lmi@unife.it (I.L.)

**Keywords:** benzothiazole derivatives, multifunctional, antioxidants, antitumor, ant-inflammatory

## Abstract

A current trend in healthcare research is to discover multifunctional compounds, able to interact with multiple biological targets, in order to simplify multi-drug therapies and improve patient compliance. The aim of this work was to outline the growing demand for innovative multifunctional compounds, achieved through the synthesis, characterisation and SAR evaluation of a series of 2-styrylbenzothiazole derivatives. The six synthesised compounds were studied for their potential as photoprotective, antioxidant, antiproliferative, and anti-inflammatory agents. In order to profile antioxidant activity against various radical species, in vitro DPPH, FRAP and ORAC assays were performed. UV-filtering activity was studied, first in solution and then in formulation (standard O/W sunscreen containing 3% synthesised molecules) before and after irradiation. Compound **BZTst6** proved to be photostable, suitable for broad-spectrum criteria, and is an excellent UVA filter. In terms of antioxidant activity, only compound **BZTst4** can be considered a promising candidate, due to the potential of the catechol moiety. Both also showed exceptional inhibitory action against the pro-inflammatory enzyme 5-lipoxygenase (LO), with IC_50_ values in the sub-micromolar range in both activated neutrophils and under cell-free conditions. The results showed that the compounds under investigation are suitable for multifunctional application purposes, underlining the importance of their chemical scaffolding in terms of different biological behaviours.

## 1. Introduction

Addressing multifactorial diseases is a formidable challenge, given the complex etiology of conditions such as cancer, Alzheimer’s disease, Parkinson’s disease, and diabetes as well as cardiovascular, central nervous system, and metabolic disorders [1,2,3,4,5]. These diseases impact multiple biological pathways, evolving over time and presenting numerous pathophysiological issues. The traditional approach of a single drug targeted at a specific site has not only proved ineffective, but has often led to drug resistance phenomena [6,7]. To effectively tackle these diseases, drug combination therapies are necessary. These therapies target individual biological changes or symptoms rather than the entire disease [8]. Although this strategy is the standard, it raises concerns about cost and patient adherence due to the complexity of managing multiple medications over time. Moreover, there are challenges in determining pharmacokinetics and the risk of adverse interactions, which may reduce efficacy and patient compliance and increase therapy costs and severe side effects [9,10]. In light of these challenges, multifunctional compounds that possess multiple biological activities within a single molecule hold promise. These compounds can deliver enhanced therapeutic effects through synergistic actions and reduced side effects. By targeting multiple points within a disease pathway, multifunctional compounds offer more effective modulation of complex diseases without a corresponding increase in adverse effects [6,11,12,13]. The recent push for the development of multifunctional compounds has gained significant interest in treating diseases lacking adequate options. Notably, skin cancer, one of the most prevalent multifactorial diseases, has the highest global incidence, accounting for one in three cancer diagnoses with over 2 million new cases annually [14].

The climate changes together with a change in the behaviour model of tanning are leading an increase in skin cancer occurrence [15,16]. UV radiation, especially in combination with pollution, is the primary etiological factor responsible for skin cancer development [17,18,19,20,21,22]. UV radiation-induced “signature” mutations contribute significantly to the development of melanoma and other skin cancers [23,24]. UV overexposure can lead to oxidative stress, where reactive oxygen species (ROS) generated by UVA damage DNA, potentially causing mispairing, mutations, and uncontrolled cell proliferation [25,26]. Melanoma cells, in particular, produce more ROS than normal melanocytes, emphasizing the significance of ROS in melanoma development [27]. UV radiation-related injuries trigger pro-inflammatory processes, such as the activation of phospholipase A_2_ (PLA_2_), increasing the liberation of free polyunsaturated fatty acids and downstream production of lipid mediators [28]. Such products, derived from lipoxygenases (LO) and cyclooxygenases (COX), play crucial roles in initiating and maintaining the inflammatory response following UV exposure, contributing to tumor progression and sustaining the photocarcinogenic process [29,30,31]. Sunscreens combined with adequate exposure in the correct hours are a good approach to protect ourselves against the above-mentioned risks [32].

Our research group is committed to developing, characterizing, and biologically evaluating novel chemical entities that exhibit multiple activities, with the ultimate goal of treating and preventing complex skin diseases such as melanoma and non-melanoma skin cancers [33,34,35]. These entities are designed to work synergistically and possess desired activities, including broad-spectrum photoprotection (280–400 nm), significant antioxidant and ROS scavenging activity, anti-inflammatory potential to counteract UV radiation’s harmful effects, and direct antiproliferative effects on specific tumor cell lines. One of our lead compounds is 2-Phenyl-1H-Benzimidazole-5-Sulphonic Acid (PBSA), which has been extensively used as a UVB filter in the sunscreen industry due to its safety and efficacy. However, PBSA lacks UVA photoprotection and antioxidant activity, necessitating its combination with other antioxidants and/or UVA filters in cosmetic formulations. Taking this into account, we have chosen PBSA as scaffold for sequential modifications (i.e., isosteric)—a strategy commonly employed in pharmaceuticals for the rational design of new drugs—evaluating their effects in order to obtain derivatives of potential therapeutic interest.

## 2. Materials and Methods

### 2.1. General

All reagents were purchased from Sigma Aldrich SRL, Milano, Italy. All solvents used were purchased from Carlo Erba Reagents SRL, Milano, Italy, and used without further purification. Silica gel plates were used to perform TLC analysis (Macherey-Nagel Poligram SIL G/UV2540.20 mm, GmbH & Co. KG Neumann-Neander-str. 6–8, Dueren, Germany) and visualized by a UV lamp with the wavelength fixed to 254 nm and/or with a solution of KMnO4 (1%). Molecular weights were determined by ESI-MS (Micromass ZMD 2000), and the values are reported as [M+H]+. IR spectra were registered with a Spectrum 100 FT-IR Spectrophotometer (PerkinElmer, Milan, Italy), and the main band is reported as cm^−1^. Melting points were determined by a Stuart melting point apparatus. ^1^H-NMR and ^13^C-NMR were registered on the VXR-200 Varian spectrometer at 200 MHz and 400 MHz, using tetramethylsilane (TMS) as an internal standard. The chemical shift of each signal is expressed as units δ (ppm) relative to the signal of deuterated solvents used (DMSO-d6 and CDCl_3_). The following abbreviations are used to designate multiplicity and assignment: s (singlet), d (doublet), t (triplet), m (multiplet), dd (double doublet), BZT (benzothiazole), and Ar (Aryl). All NMR spectra will be provided in the Supplementary Materials. UV spectrophotometric analysis was conducted on a UV-Vis spectrophotometer (UV-31 SCAN ONDA Spectrophotometer, Giorgio Bormac spectrophotometer Srl, Carpi (MO), Italy), while spectrophotometric analysis for the detection of filter parameters used a UV-Vis spectrophotometer (Shimadzu UV-2600). WW5 PMMA plates were purchased from Schonberg GmbH (Munich, Germany). Photostability of the compounds was evaluated with Atlas Suntest CPS+ solar simulator, (URAI S.p.a., Assago, Milano, Italy). The instrument used to conduct ORAC analyses was the Thermo Fluoroskan Ascent FL^®^ Microplate Fluorometer and Luminometer, with fluorescent filters (excitation wavelength: 485 nm; emission filter: 538 nm), linked to Ascent Software^®^ (version 2.1) for data control and processing. In the sample loading phase, 96-well plates with a black background were used.

### 2.2. Chemistry

#### 2.2.1. Synthesis of 2-Methylbenzo[d]thiazole (**2**)

2-Aminothiophenol (6 mL, 56.1 mmol) was solubilized in acetic anhydride (25 mL) in a 100 mL round-bottomed flask and heated to reflux conditions (140–150 °C) for 3.5 h. Reaction was monitored by TLC. The flask was then cooled, and the mixture neutralized with 0.5 M aqueous NaOH, then extracted with EtOAc (3 × 15 mL), dried over Na_2_SO_4_, and concentrated and purified by column chromatography (EtOAc: petroleum ether 0.5:9.5) to afford compound **2** in good yield (67%) as a pale-yellow oil. Analytical data are in agreement with those reported in the literature [36].

#### 2.2.2. General Procedure for the Synthesis of 2-Styrylbenzothiazoles **BZTst1** and **BZTst3**

Compound **2** (305 mg, 2.1 mmol) was placed in a screw-capped glass vial with 10–15 drops of acetic anhydride. The suitable aromatic aldehyde (3.1 mmol) was added, and the reaction mixture was stirred at 120 °C for 16–18 h. Reaction was monitored by ESI-MS. After completion, reaction was cooled to r.t. and diluted with 3 mL of MeOH: H_2_O mixture (1:2) while stirring. After neutralization with 2M aqueous NaOH, the precipitate formed was filtered and washed several times with petroleum ether, dried, and finally recrystallized from EtOH to afford target compounds.

(*E*)-2-styrylbenzo[d]thiazole (**BZTst1**): yellow crystals, yield 52.1%. M.p. 114–116 °C; IR (cm^−1^): 3059.52, 1629.29, 1444.93, 1435.02, 1314.09, 1189.20,1072.24, 957.26. ^1^H-NMR (400 MHz, CDCl3): δ(ppm) 8.01 (dd, 1H, ***J*1** = 8 Hz, ***J*2** = 0.8 Hz, BZT), 7.87 (dd, 1H, ***J*1** = 8 Hz, ***J*2** = 0.8 Hz, BZT), 7.59 (t, 1H, ***J*** = 7.8 Hz, BZT), 7.55 (d, 1H, ***Jtrans*** = 18.4 Hz, Htrans), 7.42 (d, 1H, ***Jtrans*** = 16.4 Hz, Htrans), 7.36–7.48 (m, 6H, 1H BZT + 5H Ar). ^13^C-NMR (400 MHz, CDCl3): δ(ppm) 137.83 (BZT), 135.36 (Ar), 134.25 (BZT), 129.5 (-HC=CH-), 128.96 (4C, Ar), 127.43 (Ar), 126.38 (BZT), 125.42 (BZT), 122.91 (-HC=CH-), 122.00 (BZT), 121.52 (BZT). ESI-MS[M+H]+: calculated for C_15_H_11_NS, 238.06, found, 238.20.

(*E*)-2-(4-methoxystyryl)benzo[d]thiazole (**BZTst3**): yellow-brownish solid, yield 58.9%. M.p. 144–148 °C; IR (cm^−1^): 2996.03, 2841.26, 1625.33, 1597.57, 1510.35, 1254.62, 1171.36, 1022.68, 957.26. ^1^H-NMR (400 MHz, CDCl3): δ(ppm) 7.99 (d, 1H, ***J*** = 8.4 Hz, BZT), 7.85 (d, 1H, ***J*** = 8 Hz, BZT), 7.54 (d, 2H, ***J*** = 8.4 Hz, Ar), 7.50 (d, 1H, ***Jtrans*** = 15.6 Hz, Htrans), 7.46 (t, 1H, ***J*** = 8 Hz, BZT), 7.36 (t, 1H, ***J*** = 7.6 Hz, BZT), 7.29 (d, 1H, ***Jtrans*** = 16.4 Hz, Htrans), 3.85 (s, 3H, -OCH_3_). ^13^C-NMR (400 MHz, DMSO-d6): δ(ppm) 167.31 (BZT), 160.91 (Ar), 153.96 (BZT), 137.78 (-HC=CH-), 134.33 (Ar), 129.81 (2C, Ar), 128.24 (BZT), 126.89 (BZT), 125.67 (BZT), 122.77 (BZT), 122.54 (-HC=CH-), 119.89 (BZT), 114.88 (2C, Ar), 55.76 (-OCH_3_). ESI-MS[M+H]+: calculated for C_16_H_13_NOS, 268.07, found, 268.50.

#### 2.2.3. General Procedure for the Synthesis of 2-Styrylbenzothiazoles **BZTst2**, **BZTst4–6**

Compound **2** (305.4 mg, 2.05 mmol) was placed in a screw-capped glass vial with 10–15 drops of acetic anhydride. The suitable aromatic aldehyde (3.10 mmol) was added, and the reaction stirred at 120 °C for 16–18 h. Reaction was monitored by ESI-MS. After completion, reaction was cooled to r.t. and diluted with 3 mL of MeOH: HCl 6N mixture (1:2) and stirred at r.t. for 24 h. When the acetylated byproducts were no longer detectable, reaction was stopped and neutralized with NaOH 3.4N. The solid formed was filtered on Gooch, washed several times with petroleum ether, dried and recrystallized from EtOH or EtOAc, to afford target compounds in moderate to good yields.

(*E*)-4-(2-(benzo[d]thiazol-2-yl)vinyl)phenol (**BZTst2**): light brown solid, yield 47.4%. M.p. 159–166 °C (dec.); IR (cm^−1^): 3400.79, 3000.00, 1666.96, 1577.75, 1512.33, 1437.00, 1375.55, 1286.34, 1220.92, 1167.40. ^1^H-NMR (400 MHz, DMSO-d6): δ(ppm) 9.92 (bs, 1H, -OH), 8.03 (dd, 1H, ***J*1** = 8 Hz, ***J*2** = 1 Hz, BZT), 7.91 (dd, 1H, ***J*1** = 8Hz, ***J*2** = 1Hz, BZT), 7.60 (dd, 2H, ***J*1** = 6.4 Hz, ***J*2** = 2 Hz, Ar), 7.54 (d, 1H, ***J*** = 16.4 Hz, -HC=CH-), 7.47 (t, 1H, ***J*** = 7.2 Hz, BZT), 7.38 (t, 1H, ***J*** = 7.2 Hz, BZT), 7.35 (d, 1H, ***J*** = 16.4 Hz, -HC=CH-), 6.81 (dd, 2H, ***J*1**= 6.6 Hz, ***J*2** = 2.2 Hz, Ar). ^13^C-NMR (400 MHz, DMSO-d6): δ(ppm) 167.51 (BZT), 159.52 (Ar), 153.98 (BZT), 138.26 (-HC=CH-), 134.23 (Ar), 129.96 (2C, Ar), 126.85 (BZT), 125.56 (BZT), 122.69 (-HC=CH-), 122.49 (BZT), 118.85 (BZT), 116.27 (2C, Ar). ESI-MS[M+H] +: calculated for C_15_H_11_NOS, 254.07, found, 254.62.

(*E*)-4-(2-(benzo[d]thiazol-2-yl)vinyl)benzene-1,2-diol (**BZTst4**): brown solid, yield 43.2%. M.p. 223–227 °C (dec.); IR (cm^−1^): 3440.47, 2996.03, 1599.55, 1433.03, 1282.37, 1218.94, 113.87, 933.48. ^1^H-NMR (400 MHz, DMSO-d6): δ(ppm) 9.50 (bs, 1H, -OHp), 9.11 (bs, 1H, -OHm), 8.03 (d, 1H, ***J*** = 8.4 Hz, BZT), 7.90 (d, 1H, ***J*** = 8.4 Hz, BZT), 7.47 (t, 1H, ***J*** = 8 Hz, BZT), 7.45 (d, 1H, ***Jtrans*** = 16 Hz, -Htrans), 7.37 (t, 1H, ***J*** = 7.6 Hz, BZT), 7.23 (d, 1H, ***Jtrans*** = 16 Hz, Htrans), 7.11 (d, 1H, ***J*** = 1.6 Hz, Ar), 7.04 (dd, 1H, ***J*1** = 8.4 Hz, ***J*2** = 1.6 Hz, Ar), 6.78 (d, 1H, ***J*** = 8.4 Hz, Ar). ^13^C-NMR (400 MHz, DMSO-d6): δ(ppm) 167.48 (BZT), 153.97 (BZT), 148.06 (Ar), 146.08 (Ar), 138.69 (-HC=CH-), 134.23 (Ar), 127.13 (Ar), 126.83 (Ar), 125.54 (BZT), 122.64 (BZT), 122.48 (-HC=CH-), 120.99 (BZT), 118.68 (BZT), 116.27 (Ar), 114.70 (Ar). ESI-MS[M+H] +: calculated for C_15_H_11_NO_2_S, 270.05, found, 270.27.

(*E*)-5-(2-(benzo[d]thiazol-2-yl) vinyl)-2-methoxyphenol (**BZTst5**): light brown solid, yield 60.3%. M.p. 184–187 °C; IR (cm^−1^): 3027.77, 1627.31, 1597.57, 1520.26, 1435.02, 1203.08, 1127.75, 1028.63, 945.37. ^1^H-NMR (400 MHz, DMSO-d6): δ(ppm) 9.21 (s, 1H, -OH), 8.04 (d, 1H, ***J*** = 8 Hz, BZT), 7.92 (d, 1H, ***J*** = 8.4 Hz, BZT), 7.50 (d, 1H, ***Jtrans*** = 16.4 Hz, Htrans), 7.48 (t, 1H, ***J*** = 8 Hz, BZT), 7.39 (t, 1H, ***J*** = 7.6 Hz, BZT), 7.32 (d, 1H, ***Jtrans*** = 16 Hz, Htrans), 7.16 (m, 2H, Ar), 6.96 (d, 1H, ***J*** = 8.8 Hz, Ar). ^13^C-NMR (400 MHz, DMSO-d6): δ(ppm) 167.29 (BZT), 153.96 (BZT), 149.82 (Ar), 147.18 (Ar), 138.25 (-HC=CH-), 134.32 (Ar), 128.53 (Ar), 126.87 (Ar), 125.64 (BZT), 122.73 (BZT), 122.52 (-HC=CH-), 119.68 (BZT), 114.18 (BZT), 112.52 (Ar), 56.09 (-OCH_3_). ESI-MS[M+H] +: calculated for C_16_H_13_NO_2_S, 284.07, found, 284.16.

(*E*)-4-(2-(benzo[d]thiazol-2-yl) vinyl)-2-methoxyphenol (**BZTst6**): deep brown solid, yield 42.9%. M.p. 139–144 °C; IR (cm^−1^): 2928.57, 1599.55, 1510.35, 1423.12, 1286.34, 1205.06, 1117.84, 1018.72, 963.21. ^1^H-NMR (400 MHz, DMSO-d6): δ(ppm) 9.65 (s, 1H, -OH), 8.04 (d, 1H, ***J*** = 7.6 Hz, BZT), 7.90 (d, 1H, ***J*** = 7.6 Hz, BZT), 7.53 (d, 1H, ***Jtrans*** = 16 Hz, -Htrans), 7.47 (t, 1H, ***J*** = 7.6 Hz, BZT), 7.44 (d, 1H, ***Jtrans*** = 16 Hz, Htrans), 7.38 (t, 1H, ***J*** = 8 Hz, BZT), 7.38 (d, 1H, ***J*** = 1.6 Hz, Ar), 7.15 (dd, 1H, ***J*1** = 8 Hz, ***J***2 = 1.6 Hz, Ar), 6.84 (d, 1H, ***J*** = 8.4 Hz, Ar). ^13^C-NMR (400 MHz, DMSO-d6): δ(ppm) 167.51 (BZT), 154.02 (BZT), 149.13 (Ar), 148.44 (Ar), 138.55 (-HC=CH-), 134.25 (Ar), 127.10 (Ar), 126.85 (Ar), 125.54 (BZT), 122.65 (BZT), 122.51 (-HC=CH-), 119.06 (BZT), 116.11 (BZT), 111. 22 (Ar), 56.15 (-OCH_3_). ESI-MS[M+H]+: calculated for C_16_H_13_NO_2_S, 284.07, found, 284.18.

### 2.3. Photoprotective Activity

#### 2.3.1. In Vitro Evaluation of Filtering Parameters of Compounds in Solution

The synthesized compounds were solubilised in DMSO and diluted in MeOH, and the absorbance of the 10 µg/mL solution was measured between 290–400 nm with a UV-Vis spectrophotometer (SHIMADZU UV-2600 240 V, 1 cm quartz cell at 1 nm intervals). The absorbance at wavelength λ is related to the transmittance T(λ) via the Equation (1):

A(λ) = −Log[T(λ)](1)

where T(λ) is the fraction of incident irradiance transmitted by the sample.

Then, filtering parameters (SPF, UVAPf, UVA/UVA and ʎc) were calculated by applying Equations (2)–(4) using SPF Calculator Software (SPF Calculator Software version 2.1, Shimadzu, Milan, Italy):

(2) $${\mathrm{SPF}}_{\mathrm{in}\;\mathrm{vitro}}=\frac{\int_{\lambda =290\mathrm{nm}}^{\lambda =400\mathrm{nm}}E\left(\lambda \right)\times I\left(\lambda \right)\times d\lambda }{\int_{\lambda =290\mathrm{nm}}^{\lambda =400\mathrm{nm}}E\left(\lambda \right)\times I\left(\lambda \right)\times 10^{-A_{0}\left(\lambda \right)}\times d\lambda }$$

where E(λ) is the erythema action spectrum, I (λ) is the spectral irradiance of the UV source, A_0_(λ) is the monochromatic absorbance of the test sample before UV exposure, and dλ is the wavelength step (1 nm):

(3) $$\mathrm{UVAPF}=\frac{\int_{\lambda =290\mathrm{nm}}^{\lambda =400\mathrm{nm}}P\left(\lambda \right)\times I\left(\lambda \right)\times d\lambda }{\int_{\lambda =290\mathrm{nm}}^{\lambda =400\mathrm{nm}}P\left(\lambda \right)\times I\left(\lambda \right)\times 10^{-A\left(\lambda \right)\times C}\times d\lambda }$$

where P(λ) is the persistent pigment darkening (PPD) action spectrum, C is the coefficient of adjustment, and A(λ) is the mean monochromatic absorbance of the sample after UV exposure:

(4) $$\int_{290\mathrm{nm}}^{\lambda_{c}}A\left(\lambda \right)\times d\lambda =0.9\int_{290\mathrm{nm}}^{400\mathrm{nm}}A\lambda \times d\lambda$$

where A(λ): monochromatic absorbance calculated from transmittance at wavelength λ. Critical wavelength means the wavelength at which the added absorbance equals 90% of the total absorbance.

#### 2.3.2. In Vitro Evaluation of Filtering Parameters of Sunscreen Formulations

Synthesized compounds were incorporated at 3% concentration in a suitable oil-in-water (O/W) emulsion for cosmetic use as sunscreen.

INCI: Aqua, Glycerin, Xanthan Gum, Phenoxyethanol (and) Ethylhexylglycerin, Tribehenin PEG-20 Esters, Cetearyl Alcohol, Dicaprylyl Carbonate, PPG 26 Buteth-26 (and) PEG 40 Hydrogenated Castor Oil, C12-15 Alkyl Benzoate, Sodium hydroxide (sol. 10%).

A protocol previously reported by us was adopted [33]. Briefly, 32.5 mg of each compound formulation was spread on the rough surface of 5 × 5 cm^2^ PMMA plates in the form of small drops of approximately equal mass, in triplicate. The plates were placed in the dark for 15–30 min to allow the formation of a sun-stabilised standard layer and then inserted into the instrument and measured at five different points on each plate (UV transmittance range 290–400 nm). The blank was a plate coated with 32.5 mg glycerine, chosen for its non-fluorescence and UV transparency. In addition, in vitro SPF, UVAPF and ʎc values were calculated according to Equations (2)–(4) above. To evaluate the booster effect of selected synthesized compounds, they were incorporated at 3% concentration in an oil-in-water (O/W) sunscreen formulation with fixed SPF and UVAPf values (30 and 10, respectively).

INCI: Aqua, Sodium Phytate, Alcohol, Panthenol, Glycerin, Sclerotium Gum, Xanthan Gum, Cetearyl Alcohol, Coco-Glucoside, Coco-Caprylate, Dicaprylyl Carbonate, Squalene, Bis-Ethylhexyloxyphenol Methoxyphenyl Triazine, Ethylhexyl Triazone, Diethylamino Hydroxybenzoyl Hexyl Benzoate, Tocopheryl Acetate, Benzyl Alcohol, Ethylhexylglycerin.

#### 2.3.3. Photostability

Oil-in-water (O/W) emulsions containing individual filters were spread on PMMA sheet and irradiated with a solar simulator by applying different doses of UVA equivalent to an erythema effective radiant exposure of 100 J/m^2^. Photostability was assessed by analysing the spectral transmittance, before and after irradiation, of the sunscreen thin film, measured before and after exposure from 290 to 400 nm. The residual percentages of SPF in vitro (%SPF_eff._) and UVAPF (%UVAPF_eff._) were calculated according to Equations (6) and (7), respectively. When the %SPF_eff._ and %UVAPF_eff._ are greater than or equal to 80, according to current regulations, a filter can be considered photostable [37].

### 2.4. Antioxidant Activity

#### 2.4.1. DPPH Radical Scavenging Activity

For all the compounds, 0.750 mL of a DMSO-MeOH solution mixed with 1.5 mL of DPPH solution (0.004%) in MeOH was analysed. The samples were kept in the dark at room temperature for 30 min and then analysed with a spectrophotometer by reading the absorbance at 517 nm (lambda max of the absorption spectrum of the DPPH radical). Equation (5) was used to obtain the percentage inhibition (%) of the radical:

DPPH radical-scavenging capacity (%) = [1 − (A_1_ − A_2_)/A_0_] × 100% (5)

where A_2_ is the blank (MeOH), and A_1_ and A_0_ are, respectively, the recorded absorbances of the sample and control (0.750 MeOH and 1.5 mL DPPH solution), directly proportional to the concentration of residual DPPH. An initial screening was performed by testing all compounds at a concentration of 1 mg/mL (Trolox^®^ was used as the standard); subsequently, compounds showing >90% radical inhibition were further tested to calculate their IC50 value (expressed in µg/mL). Ferulic acid was used as positive control. A linear regression was performed to calculate the sample concentration able to eliminate 50% of DPPH free radicals.

#### 2.4.2. Ferric Reducing Antioxidant Power (FRAP) Test

The FRAP test, based on a colorimetric reaction, can assess the ability of a sample to reduce ferric ions to ferrous ions when complexed with 2,4,6-trispiridyl-s-triazine (TPTZ). The FRAP solution consisted of 0.1 M acetate buffer (pH 3.6), 10 mmol/L of TPTZ in 40 mM HCl and a 20 mM solution of FeCl_3_, in a 10:1:1 ratio. A 1.9 mL volume of the FRAP solution was incubated with 0.1 mL of the sample solution at 37 °C for 10 min. The absorbance was then read with a UV-Vis spectrophotometer at a wavelength of 593 nm, which corresponds to the maximum absorbance peak of the Fe^2+^-TPTZ complex. Trolox^®^ was used as a standard, and ferulic acid was used as positive control, and the results are expressed as µmol TE/g.

#### 2.4.3. ORAC Assay

The ORAC assay was performed based on the previously modified Hong procedure [38]. The sodium salt of fluorescein (85 nM) was used as a target for free radical attack with 2,2′-azobis(2-amidinopropane) dichloride (AAPH) as a peroxyl radical generator in the final assay mixture (total volume of 0.2 mL). The test involves a calibration curve of Trolox (standard control) with different concentrations (40 to 240 μM). The compounds tested were first dissolved in DMSO:MeOH and then diluted in phosphate buffer solution pH 7.4. Fluorescence measurements were performed at 37 °C and recorded at 5-min intervals up to 30 min after the addition of AAPH. ORAC values were calculated as the difference in areas under the fluorescein quenching curves between the blank and the sample and were expressed as µmol Trolox equivalents (TE) per gram of dried sample.

### 2.5. Antiproliferative Activity

#### Cell Growth Inhibition Assay

Cell growth inhibition analyses were performed on human melanoma Colo38 cells [39] and skin HaCat keratinocytes [35]. Human melanoma Colo38 cells were kindly given by Dr. Patrizio Giacomini (Laboratory of Immunology, Regina Elena Institute, Rome, Italy). HaCat cells were obtained from Istituto Zooprofilattico Sperimentale della Lombardia e dell’Emilia-Romagna, Brescia, Italy. Cells were seeded at 40,000 cells/mL and 25,000 cells/mL, respectively. Colo38 cells were maintained in RPMI 1640, supplemented with 10% foetal bovine serum (FBS), penicillin (100 Units/mL), and streptomycin (100 µg/mL). HaCat cells were maintained in DMEM, supplemented with 10% fatal bovine serum (FBS), penicillin (100 Units/mL), streptomycin (100 µg/mL) and glutamine (2 mM); the incubation was performed at 37 °C in a 5% CO_2_ atmosphere. Compounds were dissolved in DMSO to obtain 50 mM stock solutions and diluted before cell treatment in MeOH. The tested compounds were added at serial dilutions to the cell cultures (from 0.1 to 100 µM) and incubated for 24 h further. Cells were then harvested, suspended in physiological solution, and counted with a Z2 Coulter Counter (Coulter Electronics, Hialeah, FL, USA). The number of cells/mL and the IC_50_ values were determined when untreated cells were in the log phase of cell growth. Samples of vehicles and untreated cells were included in each plate. Cisplatin was used as positive control for the HaCat cells, while N1-(4-arylidene)-1H-(2-OH-4-N(Et)2-phenyl)-[d]imidazole-2-carbohydrazide [36] was used as positive control for the Colo38 cell line.

### 2.6. Anti-Inflammatory Activity

#### 2.6.1. Lipid Mediator (LM) Formation in Primary Human Monocytes

Monocytes and polymorphonuclear leukocytes (PMNL) were freshly isolated from human blood. Human blood and leukocyte concentrates were provided by the Institute for Transfusion Medicine of the University Hospital Jena (Thuringia, Germany). The protocols for experiments with human blood and blood cells were approved by the ethical commission of the Friedrich Schiller University of Jena. Following blood density centrifugation and separation of cell populations, peripheral blood mononuclear cells (PBMC) were left to adhere for 1 h to cell culture flasks (monocyte-enriched PBMC). Non-adherent cells were washed off from the adherent monocytes, before pre-incubating monocytes (5 × 10^6^ cells/mL) with the test compounds for 10 min at 37 °C. LM formation was elicited with A23187 (2.5 µM) and CaCl2 (1 mM) over 30 min. The reaction was stopped with 1 mL of ice-cold methanol containing 10 µL of deuterium-labelled internal LM standards (200 nM d8-5S-HETE, d4-LTB_4_, d5-LXA_4_, d5-RvD2, d4-PGE_2_ and 10 µM d8-AA). LMs were extracted using Sep-Pak C18 35 cc Vac Cartridges (Waters, Milford, MA, USA) and analysed by UPLC-MS/MS as described by Pein et al. [40].

#### 2.6.2. 5-Lipoxygenase Inhibition in a Cell-Based Assay

Following blood density centrifugation and cell population separation, PMNL was recovered from the pellet after hypotonic lysis of erythrocytes. Freshly isolated PMNL (1 × 10^6^ cells/mL) in PBS buffer pH 7.4 with glucose (1 mg/mL) and CaCl_2_ (1 mM) was pre-incubated with the test compounds for 10 min at 37 °C. Then, 5-lipoxygenase (5-LO) product formation was started by addition of 2.5 μM Ca^2+^-ionophore A23187 plus 20 μM arachidonic acid. The reaction was stopped after 10 min at 37 °C with 1 mL of ice-cold methanol containing 200 ng PGB_1_ as internal standard. Major 5-LO products (LTB_4_, trans-LTB_4_, epi-trans-LTB_4_, and 5-HETE) were extracted by SPE using Sep-Pak C18 35 cc Vac Cartridges (Waters, Milford, MA, USA) and analysed by UPLC-MS/MS as described by Pein et al. [40].

#### 2.6.3. 5-Lipoxygenase Inhibition in a Cell-Free Assay

Human recombinant 5-LO was expressed and isolated as described before [41], and immediately used for activity assays after isolation. The 5-LO (0.5 µg, in 1 mL PBS pH 7.4 containing 1 mM EDTA) was pre-incubated with the test compounds for 10 min at 4 °C and pre-warmed for 30 s at 37 °C. 5-LO product formation was initiated by sequential addition of 1 mM CaCl_2_ and 20 μM arachidonic acid. After 10 min at 37 °C, the reaction was terminated by addition of 1 mL ice-cold methanol containing 200 ng PGB_1_ as internal standard. Formed 5-LO products (trans-LTB_4_, epi-trans-LTB_4_, and 5-HETE) were extracted using Sep-Pak C18 35 cc Vac Cartridges and analysed by UPLC-MS/MS as previously described [41].

#### 2.6.4. ROS Production in PMNL

Freshly isolated PMNL (1 × 10^6^ cells/mL) in PBS buffer pH 7.4 with glucose (1 mg/mL) was seeded in a black-bottomed 96-well plate (100 μL) and pre-incubated with test compounds together with the peroxide-sensitive DCF-DA (1 μg/mL) and CaCl_2_ (1 mM) in the dark under vigorous shaking. After 10 min at 37 °C, PMA (0.1 μM) was added. The fluorescence emission at 530 nm was measured after excitation at 485 nm in a thermally controlled (37 °C) NOVOstar microplate reader (BMG Lab technologies GmbH, Offenburg, Germany). Fluorescence was recorded over a period of 12.5 min after PMA addition, and values are reported as the ratio between fluorescence units at the 12.5 min timepoint of the selected sample and fluorescence units at the same timepoint of the control (PMA-stimulated PMNL without treatment).

## 3. Results and Discussion

### 3.1. Chemistry

**Scheme 1:**
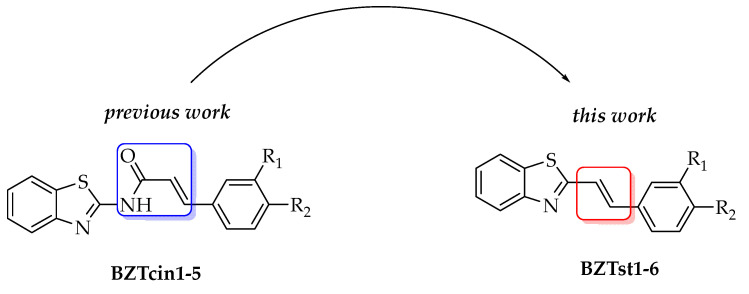
Rational design of 2-styryl-benzothiazoles **BZTst1–6**.

**Scheme 2:**
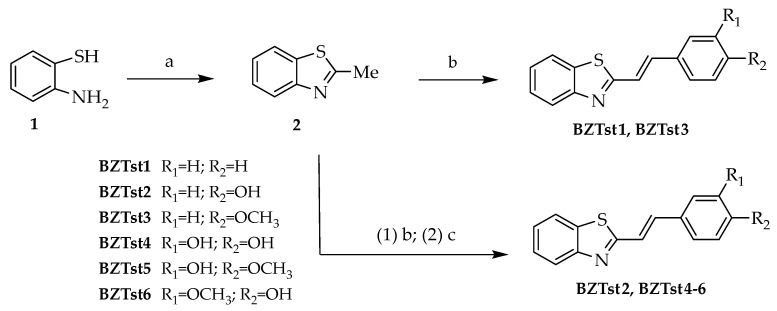
Synthetic approach for styril-benzothiazole derivatives **BZTst1–6**. Reagents and conditions: (**a**) Ac_2_O, 3.5 h, reflux; (**b**) Ar-CHO, Ac_2_O (cat.), 120 °C, 16–18 h; (**c**) HCl 6N, MeOH, 24 h, rt.

Encouraged by our previously disclosed benzothiazole cinnamate derivatives [35], we developed a small library of 2-styrylbenzothiazoles (compounds **BZTst1**–**6**) by shortening the amide-type linker system employed previously in order to maintain a minimal sequence of π-conjugated carbon atoms (Scheme 1). We planned a synthetic route for targeting 2-styrylbenzothiazoles in two stages (Scheme 2), wherein the key intermediate 2-methylbenzo[d]thiazole (**2**) was synthesized by the reported method [42] in excellent yield from 2-aminothiophenol (**1**) in refluxing acetic anhydride for 3.5 h, followed by rapid chromatography separation. The purified intermediate **2** was then reacted in neat conditions with the appropriate aromatic aldehyde, in the presence of a catalytic amount of acetic anhydride, at 120 °C for 16–18 h to afford compounds **BZTst1** and **BZTst3**. Compounds **BZTst1** and **BZTst3** were obtained in moderate to good yield after recrystallization. Compounds bearing at least one phenolic moiety underwent a further mild acidic deacetylation step to recover final products **BZTst2**, **BZTst4**, **BZTst5**, and **BZTst6** in good yield after recrystallization.

### 3.2. Antioxidant Activity

To determine the full antioxidant profile of the six synthesized compounds **BZTst1**–**6**, multiple in vitro experiments should be conducted [43]. These experiments can be categorized into two main methodologies based on the primary mechanism of radical deactivation: SET-based methods (single electron transfer) and HAT-based methods (hydrogen atom transfer). SET-based methods measure the ability of the sample to transfer a single electron to reduce a target substrate, while HAT-based methods measure the usual ability of an antioxidant to switch off free radicals by donating hydrogen [44]. All synthesized compounds were evaluated using two SET methods, the 1,1-diphenyl-2-picrylhydrazyl (DPPH) radical-scavenging activity and the ferric reducing antioxidant power (FRAP), as well as one HAT method, the oxygen radical absorbance capacity (ORAC). The results are summarized in Table 1 as micromoles of Trolox equivalents per gram of sample (µmol TE/g) for the FRAP and ORAC assays. To facilitate a better understanding of the DPPH test results, the values are reported as the inhibition percentage (IP %) of the DPPH radical at a concentration of 1 mg/mL.

The DPPH inhibition data presented in Table 1 indicate that the 2-strilbenzothiazole derivatives lacked satisfactory radical activity, with the exception of the compound **BZTst4** (3,4-dihydroxystil), which exhibited an IC_50_ value in the low micromolar range, comparable to that of the reference antioxidant compound, ferulic acid. The catechol part appears to be crucial for antioxidant activity in this test.

In the FRAP assay, the monosubstituted styryl derivatives and the disubstituted derivative **BZTst5** with a methoxy group in the para position showed poor antioxidant properties, while the disubstituted compounds with a hydroxyl group in the para position (e.g., **BZTst4**) showed significant antioxidant capacity. From the ORAC assay, the most promising candidates were the styryl derivatives **BZTst2** and **BZTst6**, both with a hydroxyl in the para position of the benzene ring: the latter showed an outstanding value of 19,027.54 ± 46.67 µmol TE/g, even surpassing ferulic acid. The differences between the DPPH/FRAP and ORAC values can be attributed to the different mechanisms involved in SET-based reactions, where the dissociation of the phenolic proton is crucial for the subsequent electron transfer, and the presence of an electron-withdrawing group (EWG) near the hydroxyl moiety facilitates this step.

### 3.3. Evaluation of In Vitro UV-Filtering Parameters

#### 3.3.1. UV-Filtering Parameters in Solution

**Figure 1:**
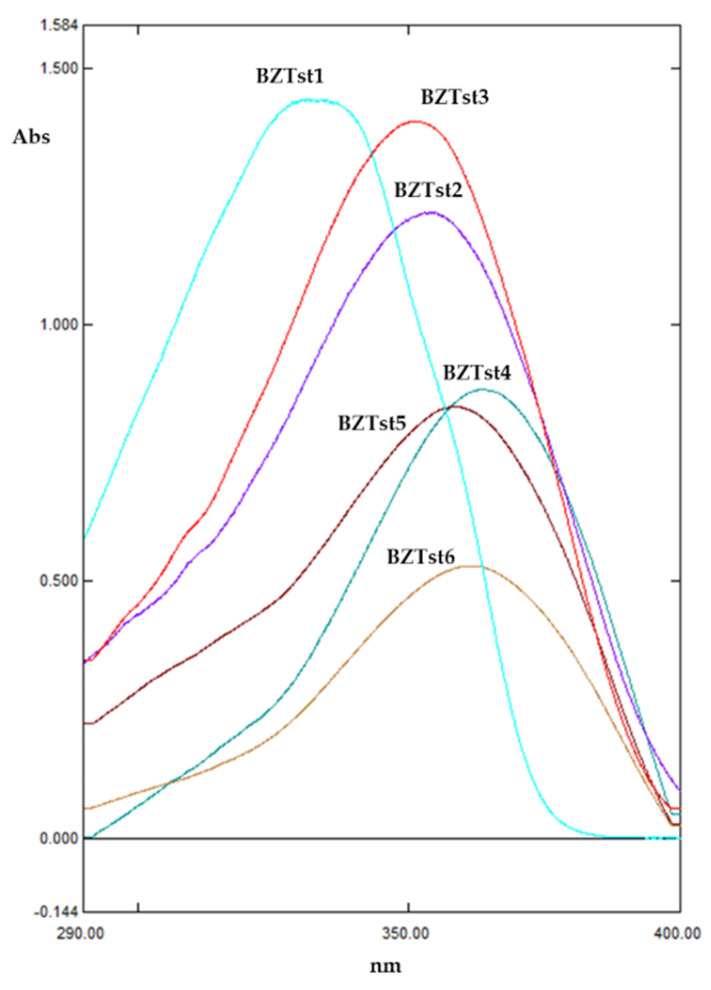
Overlaid absorbance spectra of styryl-benzothiazoles **BZTst1**–**6**. Light blue (**BZTst1**), violet (**BZTst2**), red (**BZTst3**), teal (**BZTst4**), brown (**BZTst5**), light brown (**BZTst6**).

The spectrophotometric behaviour of 2-styrylbenzothiazoles **BZTst1**–**6** underlines very distinct profiles that are closely related to the nature and position of aryl substituents (Figure 1).

The compounds **BZTst1** and **BZTst3**, respectively utilizing a naked aromatic ring or a 4-methoxy group, exhibit the highest Ɛ of the series, with values of 34,075.83 and 35,125.63, respectively (Table 2). As the number of hydroxy/methoxy groups increases, the Ɛ values decrease, with the lowest value of 16,871.66 observed for compound **BZTst6**. These findings have important implications for the filtering capacity of the compounds. Generally, it can be concluded that EWG groups in para position cause a bathochromic effect, shifting the absorption spectra of the molecule towards the UVA region, while an EDG in the same position can mildly increase the absorbance. As the number of substituents grows, the UV absorption capacity decreases, a strong hypochromic effect is observed, and ʎ_max_ moves towards higher wavelengths, making these compounds suitable for broad-spectrum protection applications. Notably, the ʎ_max_ values of derivatives **BZTst1–6** fall within the UVA region, ranging from 335.0 to 364.1 nm. This is likely due to the highly conjugated olefin system, which lowers the transition gap between the ground state and excited state, and consequently facilitates the transition upon UV absorption.

It can be concluded that, for this class of compounds, the insertion of an EDG group in *para* position can moderately increase UV absorption capacity of the candidate filter. The molar extinction coefficient was calculated with the absorbance recorded at the ʎ_max_ applying the Lambert–Beer equation.

#### 3.3.2. UV-Filtering Parameters in Formula

Aiming to create compounds with diverse biological activities, the synthesized 2-styrylbenzothiazoles were examined for potential applications in sunscreens. Each candidate was incorporated into a standard cosmetic formulation at a concentration of 3% and subjected to photoprotective efficacy testing using the Diffey–Robson method [45]. The formulation comprised an oil-in-water (O/W) emulsion suitable for sunscreen use, with each benzothiazole derivative being heat-solubilized in the oil phase and then dispersed into the emulsion using a turbo emulsifier. The mixture was then cooled, and the emulsion was spread onto polymethylmethacrylate (PMMA) plates before recording the transmittance spectra. The SPF value, UVA protection factor value (UVAPF), the UVA/UVA ratio, and the critical wavelength (λc) were determined from the transmittance spectra using SPF calculator software and are presented in Table 3. These parameters are essential for evaluating the efficacy of potential sunscreen products.

In line with the guidelines provided by the Food and Drug Administration [46], a sunscreen can be considered broad-spectrum, i.e., capable of simultaneously protecting the skin from UVA and UVB rays, when it has a ʎc value higher than 370 nm. According to this criterion, all compounds listed in Table 3 meet the afore-mentioned standard and could thus be classified as broad-spectrum sunscreen substances. As per the directives outlined by the EU [47], the minimum efficacy requirements for a sunscreen product include an SPF value of 6 (or higher), a UVA/UVB ratio of 1/3, and a critical λ (ʎc) value greater than 370 nm. Compound 19 was the only one that met the minimum SPF value required for a suitable sunscreen, but it displayed a ʎc value below 370 nm and, therefore, could not be considered for broad-spectrum sunscreen application. All other candidates fulfilled the second and third criteria but not the first. It should be noted that all compounds exhibited a UVA/UVB ratio exceeding (or close to) 1, far above the threshold value of 1/3 established by the European Commission. The benzothiazole derivatives have demonstrated the ability to absorb ultraviolet light, particularly in the UVA range, making them suitable for broad-spectrum photoprotection applications. In terms of structure–activity relationship, an increase in the number of substituents on the aromatic ring results in a progressive shift in the absorption spectra from the UVB to the UVA region. This is evident in the differences in the absorption spectra between compound **BZTst1** (2-styrylbenzothiazole) and compound **BZTst6** ((3-methoxy-4-hydroxystyryl) benzothiazole). Compound **BZTst1** possesses the highest SPF value of the series (13.88), and the absorption profile shifted towards the UVB region, as demonstrated by its UVA/UVB ratio and λc. Compound **BZTst6**, on the other hand, exhibits the characteristics of an excellent UVA filter, with the greatest UVAPF value (14.13), a UVA/UVB ratio of almost 2, and the highest λc recorded for the series (388 nm). These reported parameters are remarkable, as they are relative to a single potential UV filter at a concentration of 3%. Although compound **BZTst1** is not suitable for broad-spectrum sunscreen application, its UVB filtering capacity still surpasses that of the reference UVB filter PBSA.

#### 3.3.3. Photostability

To evaluate the effectiveness and safety profile of a sunscreen formulation, it is necessary to provide data on its photostability. Photodegradation data can be obtained by recording the pre- and post-irradiation transmittance spectra of PMMA plates with finger-coat formulations exposed to a dose of UVA capable of causing erythema. This method, established by Garoli et al. [48], is used to determine photostability. The sun protection layer’s transmission in the range of 290–400 nm is recorded before and after exposure to sunlight, following the protocol.

The residual percentages of SPF in vitro (%SPF_eff._) and UVAPf (%UVAPf_eff._) were calculated using Equations (6) and (7) and are listed in Table 4. A filter can be classified as photostable if the %SPF_eff._ is greater than or equal to 80, according to regulatory standards [37].

%SPF_eff._ = in vitro SPF_after_/in vitro SPF_before_ × 100 (6)

%UVAPf_eff._ = UVAPf_after_/UVAPf_before_ × 100 (7)

where SPF_after_ and SPF_before_ are the SPF values calculated from the software after and before solar irradiation, and UVAPf_after_ and UVAPf_before_ are the UVAPf values calculated from the software after and before solar irradiation.

Substituted compounds demonstrated a satisfactory level of %SPF_eff._. Photostability was observed with all candidates, except for derivative **BZTst1**. This compound exhibited significant photodegradation after solar irradiation, resulting in a loss of nearly half its UVA PF. Therefore, it cannot be considered photostable. The other candidates exhibited no notable changes in their absorption spectra before and after irradiation, except for compound **BZTst6**, which showed a reduction in UVA protection over solar irradiation, but still within the established photostability threshold as per the current regulations. It is worth noting that benzothiazole derivative **BZTst3** displayed an unusual increase in UVA PF after irradiation. However, this behaviour was not accompanied by a shift in ʎmax values, which could have been related to photoisomerization processes.

### 3.4. Antiproliferative Activity on Colo38 and HaCat

All synthesized compounds were tested to evaluate their possible in vitro antiproliferative efficacy against two different cell lines. The immortalized human HaCat keratin cells were employed as a control for assessing the cytotoxicity towards non-cancerous skin cells and thus determining the selectivity towards tumor cell lines. In contrast, the human melanoma Colo38 cell line was chosen as skin cancer model. Data are summarized in Table 5, and the antiproliferative activity is expressed as the IC_50_ value in µM.

The selectivity index (SI) was also calculated, and it indicates the ratio between the IC_50_ of a given compound towards the non-cancerous cell line and the IC_50_ of the same compound towards the tumor cell line.

Cisplatin was used as positive control for the HaCat cell line, while a previously evaluated benzimidazolehydrazone derivative [33] was used as positive control for the Colo38 cell line.

2-Styrylbenzothiazoles **BZTst1**, **BZTst2**, **BZTst4**, and **BZTst6** exhibited antiproliferative activity against melanoma cells; however, their IC_50_ values were relatively high, ranging from 58.06 to 85.64 µM, and their selectivity was poor, ranging from 1.07 to 1.40. Since it is suggested that an SI equal or superior to 3 is desirable in order to classify a potential anti-cancer compound [49], it can be concluded that none of these derivatives is suitable for antitumor use against Colo38 melanoma cells. In terms of structure–activity relationship, shortening the linker system between the heterocycle and the phenyl ring appeared to negatively impact the activity and selectivity of the compounds, as compared to some previously published cinnamic acid derivatives [28] which showed promising antiproliferative potential. When comparing results, compounds bearing a 3,4-dihydroxy or a 3-methoxy-4-hydroxy substitution pattern on the aromatic ring displayed the most interesting antiproliferative profile; however, the linker structure was found to be crucial for the potency of the derivatives, with cinnamate derivatives being the most effective antiproliferative candidates.

### 3.5. Anti-Inflammatory Activity

#### 3.5.1. Screening for Modulation of LM Formation in Monocytes

**Figure 2:**
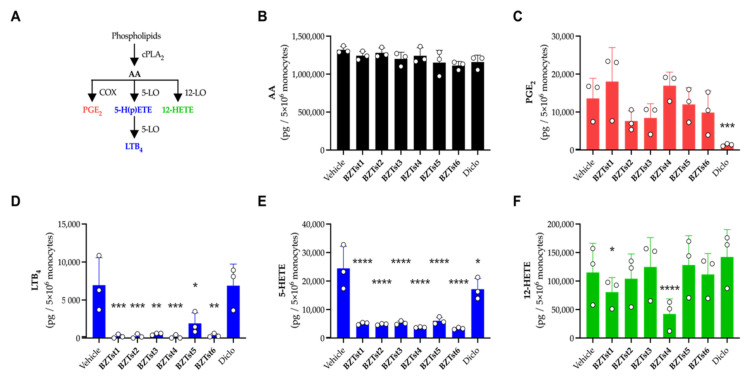
Modulation of LM formation in primary human monocytes pre-treated with vehicle (DMSO), compounds **BZTst1**–**6** (10 µM) or diclofenac (1 µM) for 10 min prior to stimulation with A23187 (2.5 µM) for 30 min. (**A**) Schematic of LM biosynthetic pathways and resulting levels of (**B**) AA, (**C**) PGE_2_, (**D**) LTB_4_, (**E**) 5-HETE, and (**F**) 12-HETE. Mean ± SD of three independent experiments. * *p* < 0.05; ** *p* < 0.01 *** *p* < 0.001; **** *p* ≤ 0.0001 (one-way ANOVA followed by Dunnett’s multiple comparison test, with a single pooled variance).

The ability to modulate LM formation was screened in primary human monocytes expressing the enzymes required for PGE_2_, LTB_4_, and 12-HETE production (Figure 2A). Cells were pre-treated with the test compounds **BZTst1**–**6** (10 µM) or the reference COX inhibitor diclofenac (1 µM) for 10 min prior to stimulation with the calcium ionophore A23187 (2.5 µM) for 30 min. While the liberation of fatty acids was not affected (Figure 2B), only diclofenac significantly downregulated PGE_2_ formation (Figure 2C). However, formation of LTB_4_ (Figure 2D) and other 5-LO products such as 5-HETE (Figure 2E) was significantly inhibited by compounds **BZTst1**–**6**. Products of other LO isoforms were not significantly affected by the test compounds (Figure 2F), with the notable exception of **BZTst1** (weak but significant inhibition of 12-HETE) and **BZTst4** (highly significant inhibition of 12-HETE).

#### 3.5.2. 5-Lipoxygenase Inhibition in Cell-Free and Cell-Based Assays

**Figure 3:**
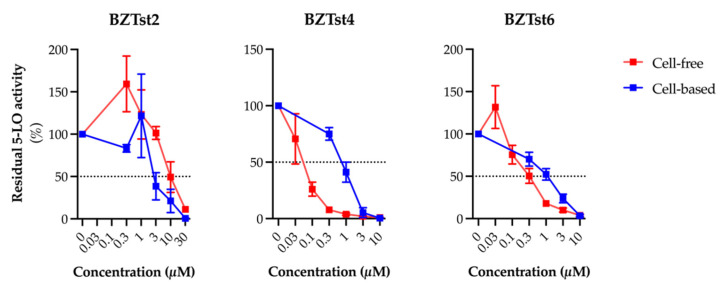
Modulation of 5-LO activity by compounds **BZTst2**, **BZTst4**, and **BZTst6** in a cell-free (isolated enzyme; red) and cell-based (primary human PMNL; blue) model. The isolated 5-LO enzyme or PMNL was pre-incubated with the indicated concentrations of compounds **BZTst2**, **BZTst4**, and **BZTst6** or vehicle for 10 min prior to stimulation with CaCl_2_ (2 mM) and AA (20 µM) (cell-free) or A23187 (2.5 µM) (cell-based) for 10 min.

The inhibitory activity of the compounds against 5-LO was further explored using another cell-based assay involving A23187-stimulated human PMNL, and a cell-free assay employing isolated human recombinant 5-LO enzyme. Initially, compounds **BZTst1–6** underwent assessment in both assays at a consistent concentration of 10 µM, in agreement with the encouraging outcomes obtained from the screening with monocytes. Results listed in Table 6 and Table 7 report interesting activities with reference to the approved 5-LO inhibitor zileuton: thus, significant inhibition was obtained only for compounds **BZTst2** (4-hydroxy), **BZTst4** (3,4-dihydroxy) and **BZTst6** (3-methoxy-4-hydroxy) that share a 4-hydroxy substitution pattern on the phenyl ring, which proved pivotal for the activity of this series. Proceeding to IC_50_ determination, compounds **BZTst4** and **BZTst6** were found to be the most potent candidates with sub-micromolar 5-LO inhibitory activities in both *cell-based* (0.70–0.95 µM) and *cell-free* (0.24–0.27 µM) assays (Figure 3). While these two compounds displayed higher activities in the *cell-free* assay, compound **BZTst2** behaved in the opposite way (IC_50_ = 2.90 µM in PMNL and 9.26 µM for isolated 5-LO). It might be possible that inhibition of 5-LO metabolite production could be due to interference with secondary biological targets. Compounds **BZTst4** and **BZTst6**, instead, showed comparable inhibitory values in both test systems that highlight a straightforward uptake from neutrophils and a direct mechanism of action. As they also express remarkable antioxidant activity, we hypothesize that they act as direct redox-active 5-LO inhibitors interfering with the redox cycle of the iron in the active site of the 5-LO enzyme [50]. Taken together, data from the two 5-LO inhibition assays clearly show a correlation between chemical structures and biological activity that allowed us to build a preliminary SAR model. Compound **BZTst4** was, for instance, able to completely turn off the enzyme’s activity at 10 µM, proving that the 3,4-dihydroxy pattern effectively inhibits 5-LO. These results are not unexpected, as many natural and synthetic inhibitors of 5-LO reported in the literature possess at least one catechol, like nordihydroguaiaretic acid (NDGA) or caffeic acid [51].

#### 3.5.3. ROS Production in Neutrophils

**Figure 4:**
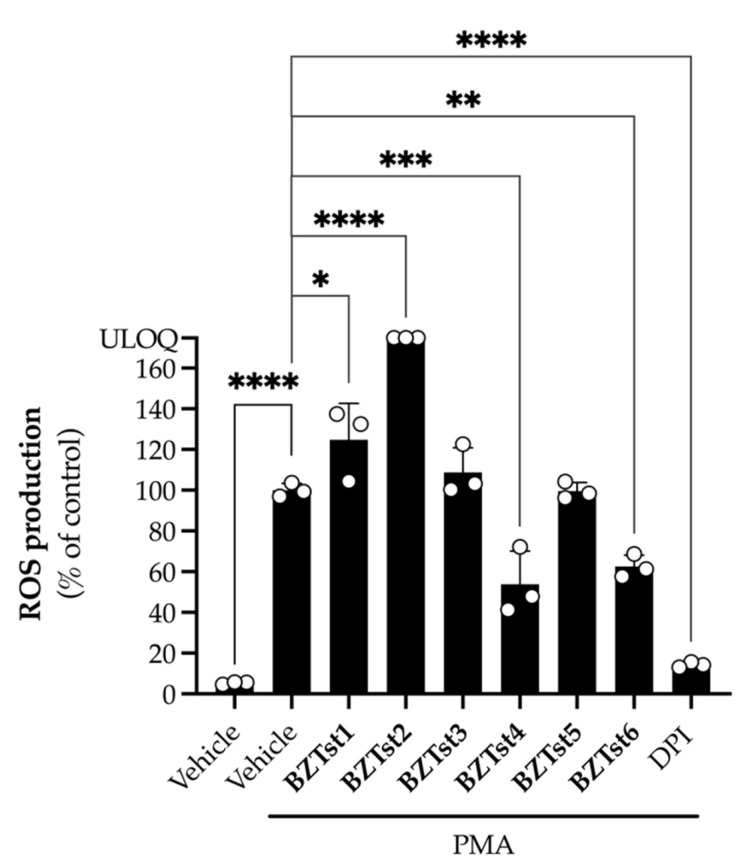
ROS production (% of control) in PMNL treated with vehicle (DMSO), compounds **BZTst1–6** (10 µM) or DPI (10 µM) prior to stimulation with PMA (10 µM). Mean ± SD of three independent experiments. * *p* < 0.05; ** *p* < 0.01 *** *p* < 0.001; **** *p* ≤ 0.0001 (one-way ANOVA followed by Dunnett’s multiple comparison test, with a single pooled variance).

Since 5-LO activity is known to be significantly influenced by redox-active substances [50] and given the notable antioxidant potential of our compound set, we opted to conduct supplementary research regarding their potential to impact intracellular ROS formation in PMNL. ROS generation was induced by phorbol-12-myristate-13-acetate (PMA), a widely recognized agent for stimulating ROS production in neutrophils and enhancing endogenous superoxide formation [52]. Diphenyleneiodonium (DPI, 10 µM), a NADPH oxidase inhibitor, served as a positive control. Through this assay, we could assess the potential interaction of our compounds with intracellular ROS production and their capacity to act as radical scavengers within a relevant cellular context. Furthermore, the outcomes provided additional insights into their inhibitory mechanism and helped to determine whether they act directly or non-specifically, as there is evidence that an impact on the ROS levels in PMNL could be translated to modulation of 5-LO activity in the cellular environment [53]. Results listed in Table 8 and Figure 4 clearly show that only compounds **BZTst4** and **BZTst6** are able to partially (<50%) lower ROS production in PMA-stimulated PMNL, while compounds **BZTst1**, **BZTst3** and **BZTst5** do not display any significant interference. With those data in hand, together with 5-LO inhibition assay outcomes, a direct inhibition of the enzyme stands as the best fitting option regarding the mechanism of action of compounds **BZTst4** and **BZTst6**. It should be noted that compound **BZTst2** provokes an unexpected rise in the ROS level in PMA-stimulated PMNL; this unusual outcome was observed in the same experiment with unstimulated PMNL (without PMA) and with PMA alone; the absence of a significant increase in ROS levels in both cases led us to conclude that the triggering of a secondary biological pathway from the combination of PMA and compound **BZTst2**, inside the cellular environment, could be responsible for the ROS production increase. This phenomenon was not further explored because such investigation was beyond the scope of this work.

## 4. Conclusions

The scope of this work was to outline the growing request for innovative multifunctional compounds able to display appreciable biological actions, achieved through the synthesis and characterization of a discrete set of 2-styrylbenzothiazole derivatives. These compounds were therefore investigated for their potential as photoprotective, antioxidant, antiproliferative, and anti-inflammatory agents. Several compounds proved suitable for multifunctional application purposes, with the chemical scaffold showing promise in exhibiting a range of biological behaviours. In particular, we found candidates for UV-filtering and photoprotection applications, as compound **BZTst6** proved to be photostable, eligible for broad-spectrum criteria and an excellent UVA filter. In terms of antioxidant activity, only compound **BZTst4** can be considered a promising candidate, owing its potential to the catechol moiety. Both compounds showed outstanding inhibitory effects towards the pro-inflammatory enzyme 5-LO, with IC_50_ values in the sub-micromolar range in both activated PMNL and in cell-free conditions. While compound **BZTst2** also exhibited anti-inflammatory activity, 5-LO inhibition was more pronounced in the PMNL cell-based assay than in the cell-free test system. It is conceivable that the inhibition of cellular 5-LO product formation may result from interference with secondary biological pathways. As far as compounds **BZTst4** and **BZTst6** are concerned, the low IC_50_ values recorded in the cell-free assay, the absence of cytotoxic effects on PMNL and the limited inhibition of ROS production in PMA-stimulated PMNL lead us to infer that the predominant mechanism of action may resemble that of selective redox-type inhibitors, capable of reducing the ferric ion within the 5-LO enzyme pocket. Docking studies may help to unravel the biological behaviour of these active derivatives. We demonstrated effects of a 3,4-hydroxyphenyl and 3-methoxy-4-hydroxyphenyl moiety to a 2-styrylbenzothiazole scaffold in producing candidates with enhanced biological activities for multifunctional applications. More investigations are underway to explore the pharmaceutical and cosmetic capabilities of these candidates, including, but not limited to, safety assessment for sunscreening purposes, skin penetration evaluation, and pharmacokinetic/pharmacodynamic profile outlining.

## Figures and Tables

**Table 1:** Antioxidant activity of 2-styrylbenzothiazoles **BZTst1**–**6**. Values in brackets indicate sample concentration (µg/mL) able to scavenge 50% of the DPPH radical (IC_50_).

Compound	Ar 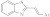	DPPH ^a^ Inhibition %	FRAP ^a^ (µmolTE/g)	ORAC ^a^ (µmolTE/g)
**BZTst1**	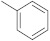	<LOQ ^b^	331.15 ± 6.29	<LOQ ^b^
**BZTst2**	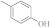	26.70 ± 0.83	1122.44 ± 25.14	11,614.64 ± 1184.06
**BZTst3**	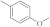	<LOQ ^b^	178.71 ± 8.17	1077.84 ± 98.52
**BZTst4**	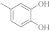	88.54 ± 0.47 (10.46 ± 0.27)	5673.33 ± 86.42	<LOQ ^b^
**BZTst5**	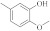	46.88 ± 0.60	788.09 ± 14.46	<LOQ ^b^
**BZTst6**	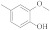	18.86 ± 0.11 ^c^	3262.22 ± 7.86	19,027.54 ± 46.67
**Ferulic Acid**	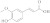	(9.90 ± 0.70)	6872.60 ± 10.70	15,906.40 ± 14.20

^a^ Each value is the mean of at least three independent experiments (mean ± SD). ^b^ Below limit of quantification. ^c^ Staining formation observed.

**Table 2:** Spectral data from spectrophotometric analysis of benzothiazole derivatives **BZTst1–6.**

Compound	ʎ_max_ (nm)	Ɛ (dm^3^ mol^−1^ cm^−1^)
**BZTst1**	335.0	34,075.83
**BZTst2**	354.4	30,835.44
**BZTst3**	351.0	35,125.63
**BZTst4**	363.3	22,070.71
**BZTst5**	358.0	22,513.37
**BZTst6**	361.2	16,871.66
**PBSA**	**304.5**	**23,881.86**

**Table 3:** Photoprotective parameters for benzothiazole derivatives **BZTst1**–**6** at 3% in O/W sunscreen formulation.

Compound	SPF	UVAPF	UVA/UVB	ʎc ^a^ (nm)
**BZTst1**	13.88	7.08	0.96	364
**BZTst2**	2.59	5.69	1.46	384
**BZTst3**	2.26	3.07	1.59	378
**BZTst4**	3.87	7.65	1.47	389
**BZTst5**	2.91	2.70	0.93	384
**BZTst6**	3.43	14.13	1.96	388
**PBSA**	1.91	1.13	0.67	357

^a^ Wavelength at which the integral of the spectral absorbance curve reaches 90% of the area under the curve from 290 to 400 nm.

**Table 4:** Photostability parameters of 2-styrylbenzothiazoles **BZTst1**–**6**.

Compound	%SPF_eff._	%UVAPf_eff._
**BZTst1**	99.14	54.38
**BZTst2**	104.43	95.96
**BZTst3**	103.21	152.12
**BZTst4**	104.02	101.57
**BZTst5**	100.00	106.30
**BZTst6**	93.59	80.75
**PBSA**	103.66	100.00

**Table 5:** Antiproliferative activity of benzothiazole derivatives on Colo-38 (human melanoma cells) and HaCat (immortalized human fibroblasts).

Compound	Colo38 (IC_50_, µM) ^a^	HaCat (IC_50_, µM) ^a^	SI
**BZTst1**	85.64 ± 2.76	>100	>1.17
**BZTst2**	79.83 ± 4.70	85.78 ± 17.88	1.07
**BZTst3**	>100	> 100	-
**BZTst4**	58.06 ± 12.10	80.19 ± 24.70	1.38
**BZTst5**	>100	85.96 ± 40.73	<1
**BZTst6**	71.53 ± 14.17	> 100	>1.40
**Cisplatin**	NT ^b^	2.00 ± 0.40	-
**[Cmp 13] ^c^**	0.70 ± 0.06	NT ^b^	-

^a^ Each value is the mean of at least three independent experiments (mean ± SD). ^b^ Not tested. ^c^ N^1^-(4-arylidene)-1H-(2-OH-4-N(Et)_2_-phenyl)-[*d*]imidazole-2-carbohydrazide [23].

**Table 6:** 5-LO inhibition values, expressed as residual enzymatic activity (%) relative to vehicle at 10 µM of test compound in activated PMNL, and respective IC_50_ values.

Cmp	5-LO Residual Activity (PMNL, 10 µM) ^a^	5-LO PMNL IC_50_ (µM) ^b^	95% CI ^c^
**BZTst1**	48.68 ± 10.21	-	-
**BZTst2**	22.89 ± 14.63	2.90	[1.3, 11.4] ^e^
**BZTst3**	75.26 ± 19.73	-	-
**BZTst4**	**0.79 ± 0.49**	**0.70**	[0.55, 0.88]
**BZTst5**	73.95 ± 25.81	-	-
**BZTst6**	**7.11 ± 4.90**	**0.95**	[0.70, 1.28]
Zileuton ^d^	31.26 ± 12.69	-	-

^a^ % relative to vehicle (DMSO). ^b^ IC_50_ values (µM) are the mean of at least three independent experiments. ^c^ CI: confidence interval for IC_50_ values. ^d^ 3 µM. ^e^ [Inhibitor] vs. normalized response.

**Table 7:** 5-LO inhibition values, expressed as residual enzymatic activity (%) relative to vehicle, at 10 µM in a cell-free environment, and respective IC_50_ values for selected compounds.

Cmp	5-LO Residual Activity (Cell-Free, 10 µM) ^a^	5-LO Cell-Free IC_50_ (µM) ^b^	95% CI ^c^
**BZTst1**	72.02 ± 20.43	-	-
**BZTst2**	47.32 ± 20.18	9.26	[4.88, 18.94]
**BZTst3**	46.73 ± 28.47	-	-
**BZTst4**	**6.85 ± 3.72**	**0.05**	[0.03, 0.06]
**BZTst5**	34.82 ± 18.39	-	-
**BZTst6**	**6.84 ± 3.38**	**0.33**	[0.17, 0.69]
Zileuton ^d^	28.64 ± 7.38	-	-

^a^ % relative to vehicle (DMSO). ^b^ IC_50_ values (µM) are the mean of at least three independent experiments. ^c^ CI: confidence interval for IC_50_ values. ^d^ 3 µM.

**Table 8:** ROS production (% of control) in PMA-stimulated PMNL at 10 µM. Mean ± SD of at least three independent experiments.

Compound	ROS Production (% of Control)
**BZTst1**	124.78 ± 17.88
**BZTst2**	**>150%** ^a^
**BZTst3**	108.70 ± 12.15
**BZTst4**	53.81 ± 16.30
**BZTst5**	99.62 ± 4.14
**BZTst6**	62.50 ± 5.62
**DPI**	16.44 ± 2.14

^a^ Plateau reached before measurement timepoint.

## Data Availability

Data are contained within the article.

## Supplementary Materials

The following supporting information can be downloaded at: https://www.mdpi.com/article/10.3390/antiox13101196/s1, Figure S1: ^1^H-NMR spectrum of compound **BZTst1**; Figure S2: ^1^H-NMR spectrum of compound **BZTst2**; Figure S3: ^1^H-NMR spectrum of compound **BZTst3**; Figure S4: ^1^H-NMR spectrum of compound **BZTst4**; Figure S5: ^1^H-NMR spectrum of compound **BZTst5**; Figure S6: ^1^H-NMR spectrum of compound **BZTst6**; Figure S7: ^13^C-NMR spectrum of compound **BZTst1**; Figure S8: ^13^C-NMR spectrum of compound **BZTst2**; Figure S9: ^3^C-NMR spectrum of compound **BZTst3**; Figure S10: ^3^C-NMR spectrum of compound **BZTst4**; Figure S11: ^3^C-NMR spectrum of compound **BZTst5**; Figure S12: ^3^C-NMR spectrum of compound **BZTst6**.

## References

[1] Graham F., Brady G.F., Kwan R., Bragazzi Cunha J., Jared S., Elenbaas J.S., Omary M.B. (2018). Lamins and Lamin Associated Proteins in Gastrointestinal Health and Disease. Gastroenterology.

[2] Singh H., Agrawal D.K. (2022). Recent advances in the development of active hybrid molecules in the treatment of cardiovascular diseases. Bioorg. Med. Chem..

[3] Kumar N., Kumar V., Anand P., Kumar V., Dwivedi A.R., Kumar V. (2022). Advancements in the development of multi-target directed ligands for the treatment of Alzheimer’s disease. Bioorg. Med. Chem..

[4] Duarte C.W., Vaughan L.K., Mark Beasley T., Tiwari H.K. (2014). Multifactorial Inheritance and Complex Diseases Reference Module. Biomedical Sciences.

[5] Storr T. (2021). Multifunctional compounds for the treatment of Alzheimer’s disease. Can. J. Chem..

[6] Bansal Y., Silakari O. (2014). Multifunctional Compounds: Smart Molecules for Multifactorial Diseases. Eur. J. Med. Chem..

[7] Makhoba X.H., Viegas C., Mosa R.A., Viegas F.P.D., Pooe O.J. (2020). Potential Impact of the Multi-Target Drug Approach in the Treatment of Some Complex Diseases. Drug Des. Devel. Ther..

[8] Keith C.T., Borisy A.A., Stockwell B.R. (2005). Multicomponent Therapeutics for Networked Systems. Nat. Rev. Drug Discov..

[9] Hohl C.M., Dankoff J., Colacone A., Afilalo M. (2001). Polypharmacy, Adverse Drug-Related Events, and Potential Adverse Drug Interactions in Elderly Patients Presenting to an Emergency Department. Ann. Emerg. Med..

[10] Reddy A.S., Zhang S. (2013). Polypharmacology: Drug Discovery for the Future. Expert Rev. Clin. Pharmacol..

[11] Morphy R., Kay C., Rankovic Z. (2004). From Magic Bullets to Designed Multiple Ligands. Drug Discov. Today.

[12] Morphy R., Rankovic Z. (2005). Designed Multiple Ligands. An Emerging Drug Discovery Paradigm. J. Med. Chem..

[13] Cavalli A., Bolognesi M.L., Minarini A., Rosini M., Tumiatti V., Recanatini M., Melchiorre C. (2008). Multi-Target-Directed Ligands to Combat Neurodegenerative Diseases. J. Med. Chem..

[14] Radiation: Ultraviolet (UV) Radiation and Skin Cancer. https://www.who.int/news-room/questions-and-answers/item/radiation-ultraviolet-(uv)-radiation-and-skin-cancer.

[15] Blashill A.J., Nogg K., Aguilar R.A.C., Roesch S., Brady J., Corliss H.L., Pagoto S., Wells K.J. (2024). Skin cancer risk behaviors in sexual minority men: A mixed methods approach. Health Psychol..

[16] Kartsagoulis A., Faust A., Carroll J., Delano A., Healey R., Lautato P., Salo M., Li H., Chen Y., Lu P. (2024). Molecular mechanisms of the effects of mulberry (*Morus Rubra*) extracts on UV-radiation and oxidative stress-induced skin cell damage. Physiology.

[17] Rhee J.S., Alex Matthews B., Neuburg M., Logan B.R., Burzynski M., Nattinger A.B. (2007). The Skin Cancer Index: Clinical Responsiveness and Predictors of Quality of Life. Laryngoscope.

[18] Tang X., Yang T., Yu D., Xiong H., Zhang S. (2024). Current insights and future perspectives of ultraviolet radiation (UV) exposure: Friends and foes to the skin and beyond the skin. Environ. Int..

[19] Bernard J.J., Gallo R.L., Krutmann J. (2019). Photoimmunology: How ultraviolet radiation affects the immune system. Nat. Rev. Immunol..

[20] Kamari F., Hallaj S., Dorosti F., Alinezhad F., Taleschian-Tabrizi N., Farhadi F., Aslani H. (2019). Phototoxicity of environmental radiations in human lens: Revisiting the pathogenesis of UV-induced cataract. Graefes Arch. Clin. Exp. Ophthalmol..

[21] Ahmed B., Qadir M.I., Ghafoor S. (2020). Malignant melanoma: Skin cancer-diagnosis, prevention, and treatment. Crit. Rev. Eukaryot. Gene Expr..

[22] Modenese A., Korpinen L., Gobba F. (2018). Solar radiation exposure and outdoor work: An underestimated occupational risk. Int. J. Environ. Res. Public Health.

[23] Leiter U., Keim U., Garbe C. (2020). Epidemiology of skin cancer: Update. Adv. Exp. Med. Biol..

[24] Seebode C., Lehmann J., Emmert S. (2016). Photocarcinogenesis and skin cancer prevention strategies. Anticancer Res..

[25] Meyskens F.L., Farmer P., Fruehauf J.P. (2001). Redox Regulation in Human Melanocytes and Melanoma. Pigment Cell Res..

[26] Rünger T.M., Kappes U.P. (2008). Mechanisms of Mutation Formation with Long-Wave Ultraviolet Light (UVA). Photodermatol. Photoimmunol. Photomed..

[27] Kamiński K., Kazimierczak U., Kolenda T. (2022). Oxidative Stress in Melanogenesis and Melanoma Development. Contemp. Oncol..

[28] Chen X., Gresham A., Morrison A., Pentland A.P. (1996). Oxidative Stress Mediates Synthesis of Cytosolic Phospholipase A2 after UVB Injury. Biochim. Biophys. Acta BBA—Lipids Lipid Metab..

[29] Krutmann J. (2006). The Interaction of UVA and UVB Wavebands with Particular Emphasis on Signalling. Prog. Biophys. Mol. Biol..

[30] McMillan T.J., Leatherman E., Ridley A., Shorrocks J., Tobi S.E., Whiteside J.R. (2008). Cellular Effects of Long Wavelength UV Light (UVA) in Mammalian Cells. J. Pharm. Pharmacol..

[31] Rhodes L.E., Gledhill K., Masoodi M., Haylett A.K., Brownrigg M., Thody A.J., Tobin D.J., Nicolaou A. (2009). The Sunburn Response in Human Skin Is Characterized by Sequential Eicosanoid Profiles That May Mediate Its Early and Late Phases. FASEB J. Off. Publ. Fed. Am. Soc. Exp. Biol..

[32] Sieniawska D., Proszowska P., Madoń M., Kotowicz Z., Orzeł A., Pich-Czekierda A., Sieniawska J. (2024). Ultraviolet-Protective Clothing and Sunscreen: Sun-Protection for Healthy Skin. J. Educ. Health Sport..

[33] Baldisserotto A., Demurtas M., Lampronti I., Tacchini M., Moi D., Balboni G., Pacifico S., Vertuani S., Manfredini S., Onnis V. (2020). Synthesis and Evaluation of Antioxidant and Antiproliferative Activity of 2-Arylbenzimidazoles. Bioorganic Chem..

[34] Djuidje E.N., Barbari R., Baldisserotto A., Durini E., Sciabica S., Balzarini J., Liekens S., Vertuani S., Manfredini S. (2022). Benzothiazole Derivatives as Multifunctional Antioxidant Agents for Skin Damage: Structure-Activity Relationship of a Scaffold Bearing a Five-Membered Ring System. Antioxidants.

[35] Barbari R., Tupini C., Durini E., Gallerani E., Nicoli F., Lampronti I., Baldisserotto A., Manfredini S. (2022). Design, Synthesis and Evaluation of New Multifunctional Benzothiazoles as Photoprotective, Antioxidant and Antiproliferative Agents. Molecules.

[36] Hati S., Sen S. (2017). Cerium Chloride Catalyzed, 2-Iodoxybenzoic Acid Mediated Oxidative Dehydrogenation of Multiple Heterocycles at Room Temperature. Eur. J. Org. Chem..

[37] Hojerová J., Medovcíková A., Mikula M. (2011). Photoprotective Efficacy and Photostability of Fifteen Sunscreen Products Having the Same Label SPF Subjected to Natural Sunlight. Int. J. Pharm..

[38] Pessina F., Marazova K., Ninfali P., Avanzi L., Manfredini S., Sgaragli G. (2004). In Vitro Neuroprotection by Novel Antioxidants in Guinea-Pig Urinary Bladder Subjected to Anoxia-Glucopenia/Reperfusion Damage. Naunyn. Schmiedebergs Arch. Pharmacol..

[39] Baldisserotto A., Buso P., Radice M., Dissette V., Lampronti I., Gambari R., Manfredini S., Vertuani S. (2018). Moringa Oleifera Leaf Extracts as Multifunctional Ingredients for “Natural and Organic” Sunscreens and Photoprotective Preparations. Molecules.

[40] Pein H., Ville A., Pace S., Temml V., Garscha U., Raasch M., Alsabil K., Viault G., Dinh C.-P., Guilet D. (2018). Endogenous Metabolites of Vitamin E Limit Inflammation by Targeting 5-Lipoxygenase. Nat. Commun..

[41] Fischer L., Szella D., Rådmark O., Steinhilber D., Werz O. (2003). Phosphorylation- and stimulus-dependent inhibition of cellular 5-lipoxygenase activity by nonredox-type inhibitors. FASEB J..

[42] Katritzky A.R., Rees C.W. (1984). Comprehensive Heterocyclic Chemistry.

[43] Alam M.N., Bristi N.J., Rafiquzzaman M. (2013). Review on in Vivo and in Vitro Methods Evaluation of Antioxidant Activity. Saudi Pharm. J..

[44] Gulcin İ. (2020). Antioxidants and Antioxidant Methods: An Updated Overview. Arch. Toxicol..

[45] Diffey B.L., Robson J. (1989). A New Substrate to Measure Sunscreen Protection Factors throughout the Ultraviolet Spectrum. J. Soc. Cosmet. Chem..

[46] Sunscreen Drug Products for Over-the-Counter Human Use. Federal Register. https://www.federalregister.gov/documents/2019/02/26/2019-03019/sunscreen-drug-products-for-over-the-counter-human-use.

[47] Verheugen G. (2006). Commission Recommendation of 22 September 2006 on the Efficacy of Sunscreen Products and the Claims Made Relating Thereto. Off. J. Eur. Union.

[48] Garoli D., Pelizzo M.G., Bernardini B., Nicolosi P., Alaibac M. (2008). Sunscreen Tests: Correspondence between in Vitro Data and Values Reported by the Manufacturers. J. Dermatol. Sci..

[49] Indrayanto G., Putra G.S., Suhud F., Al-Majed A.A. (2021). Chapter Six—Validation of in-Vitro Bioassay Methods: Application in Herbal Drug Research. Profiles of Drug Substances, Excipients and Related Methodology.

[50] Werz O., Steinhilber D. (2005). Development of 5-Lipoxygenase Inhibitors--Lessons from Cellular Enzyme Regulation. Biochem. Pharmacol..

[51] Werz O. (2007). Inhibition of 5-Lipoxygenase Product Synthesis by Natural Compounds of Plant Origin. Planta Med..

[52] Huang R., Zhao L., Chen H., Yin R.-H., Li C.-Y., Zhan Y.-Q., Zhang J.-H., Ge C., Yu M., Yang X.-M. (2014). Megakaryocytic Differentiation of K562 Cells Induced by PMA Reduced the Activity of Respiratory Chain Complex IV. PLoS ONE.

[53] Weitzel F., Wendel A. (1993). Selenoenzymes Regulate the Activity of Leukocyte 5-Lipoxygenase via the Peroxide Tone. J. Biol. Chem..

