# Supplementary material for: Synthesis and Biological Activity Assessment of 2-Styrylbenzothiazoles as Potential Multifunctional Therapeutic Agents
Source: Antioxidants (Basel). 2024 Oct 1;13(10):1196. doi: 10.3390/antiox13101196 (PMC11504387; doi:10.3390/antiox13101196)
Supplement: Supplementary file 1 [file antioxidants-13-01196-s001.zip › antioxidants-3208706-supplementary.pdf]

Supplementary Materials

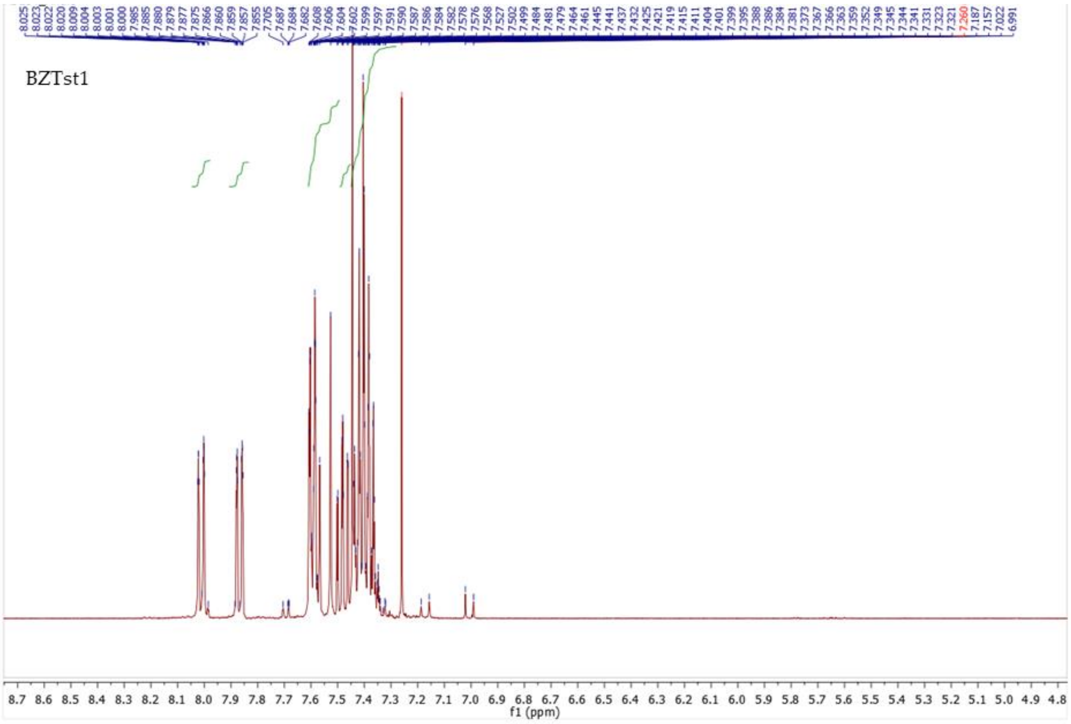

Figure S1. ^{1}H-NMR spectrum of compound **BZTst1.**

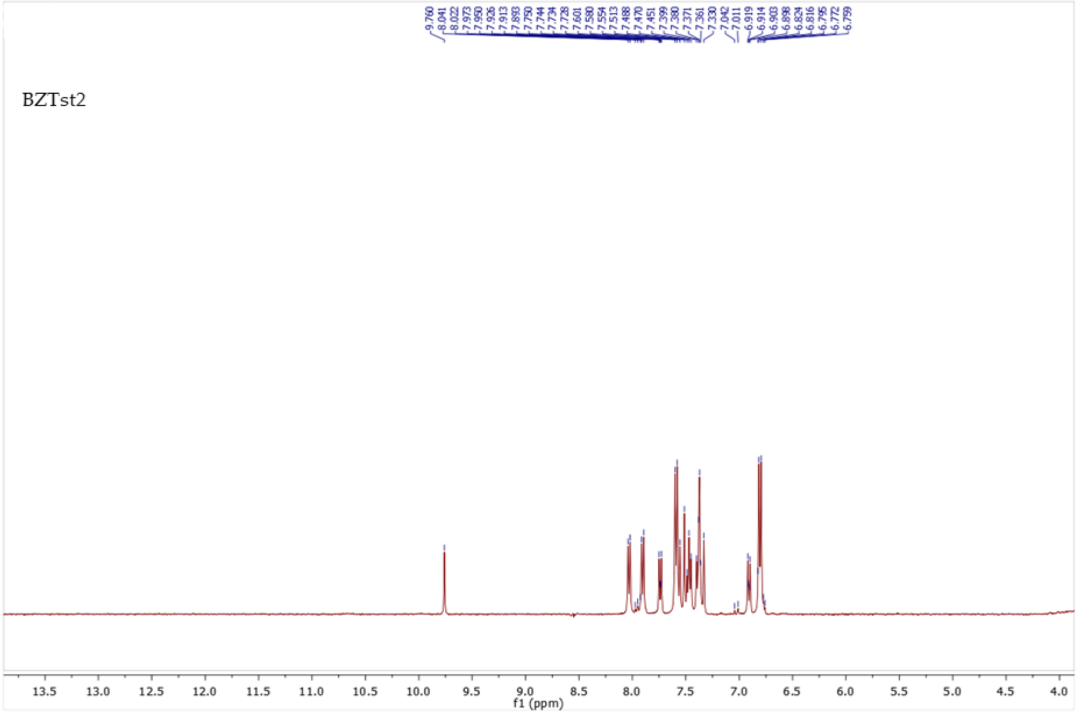

Figure S2. ^{1}H-NMR spectrum of compound **BZTst2**.

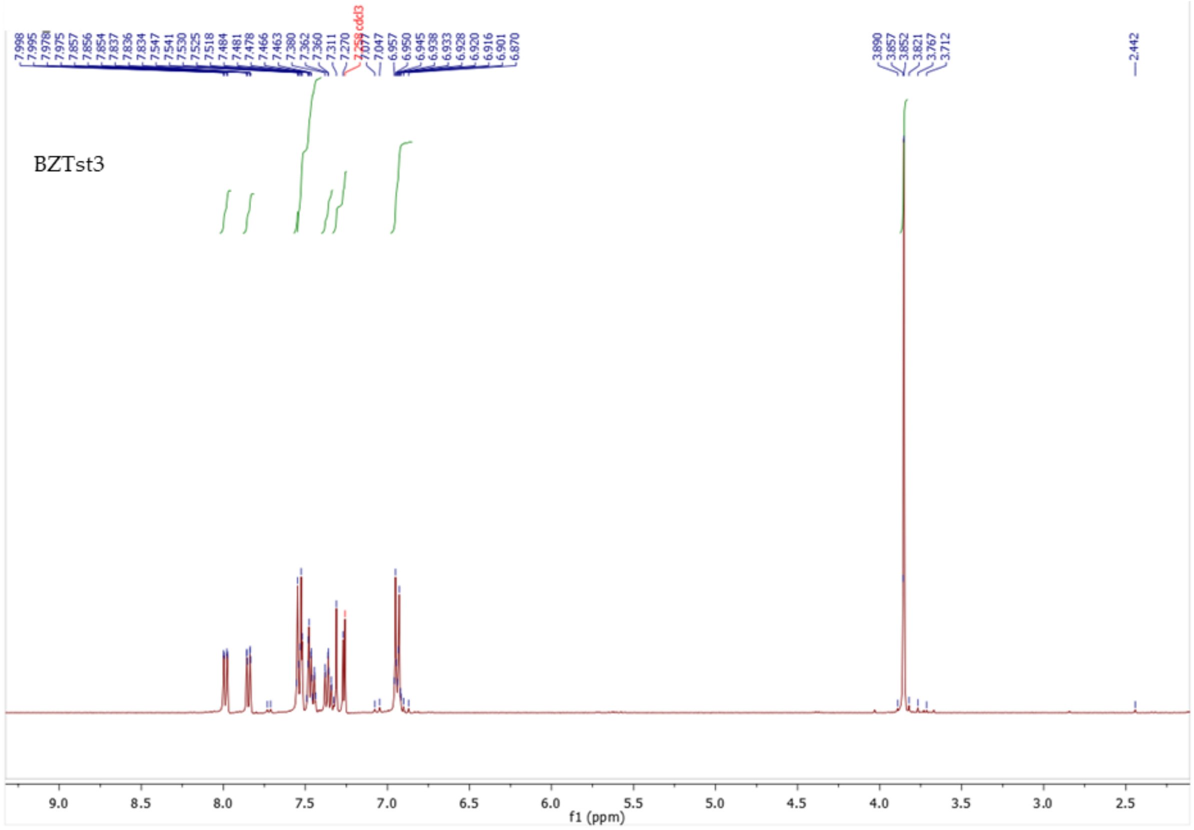

Figure S3. ^{1}H-NMR spectrum of compound **BZTst3.**

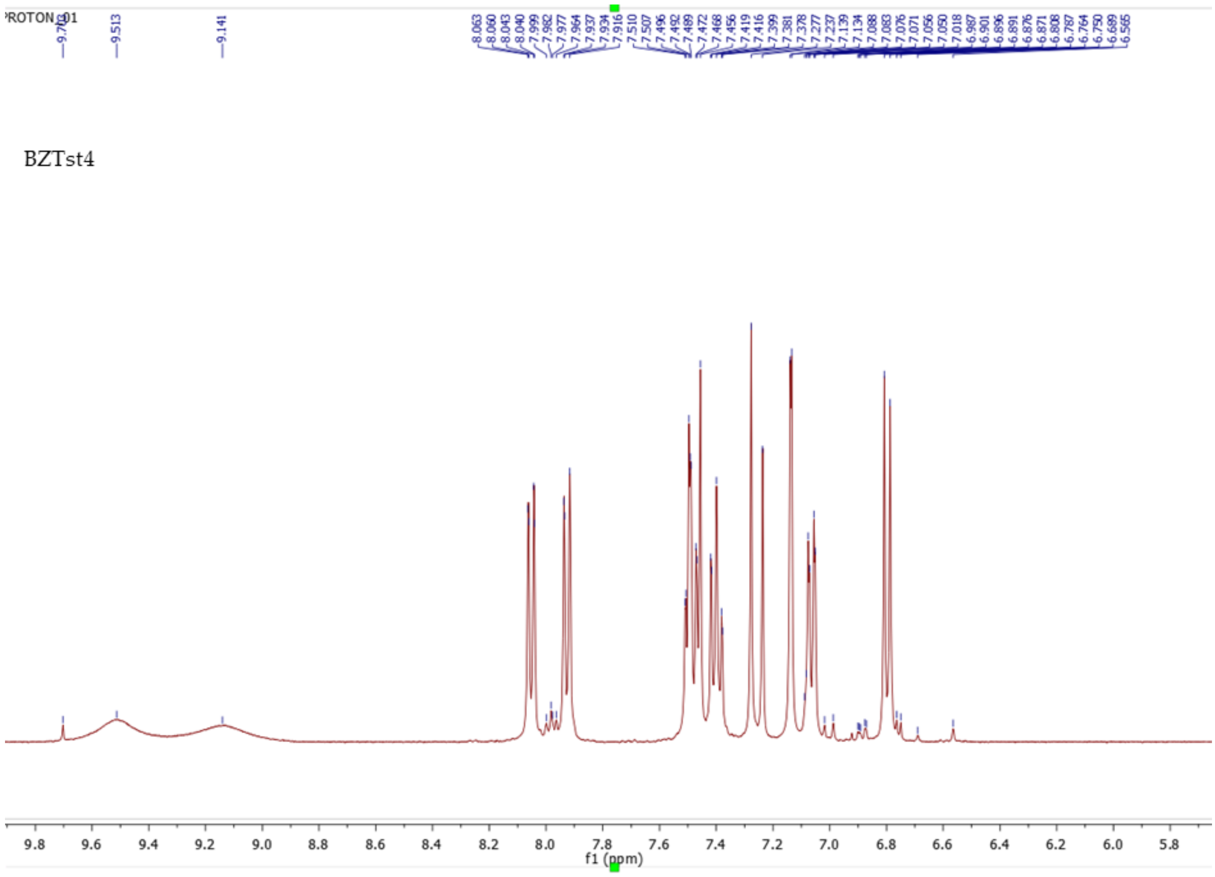

Figure S4. ^{1}H-NMR spectrum of compound **BZTst4.**

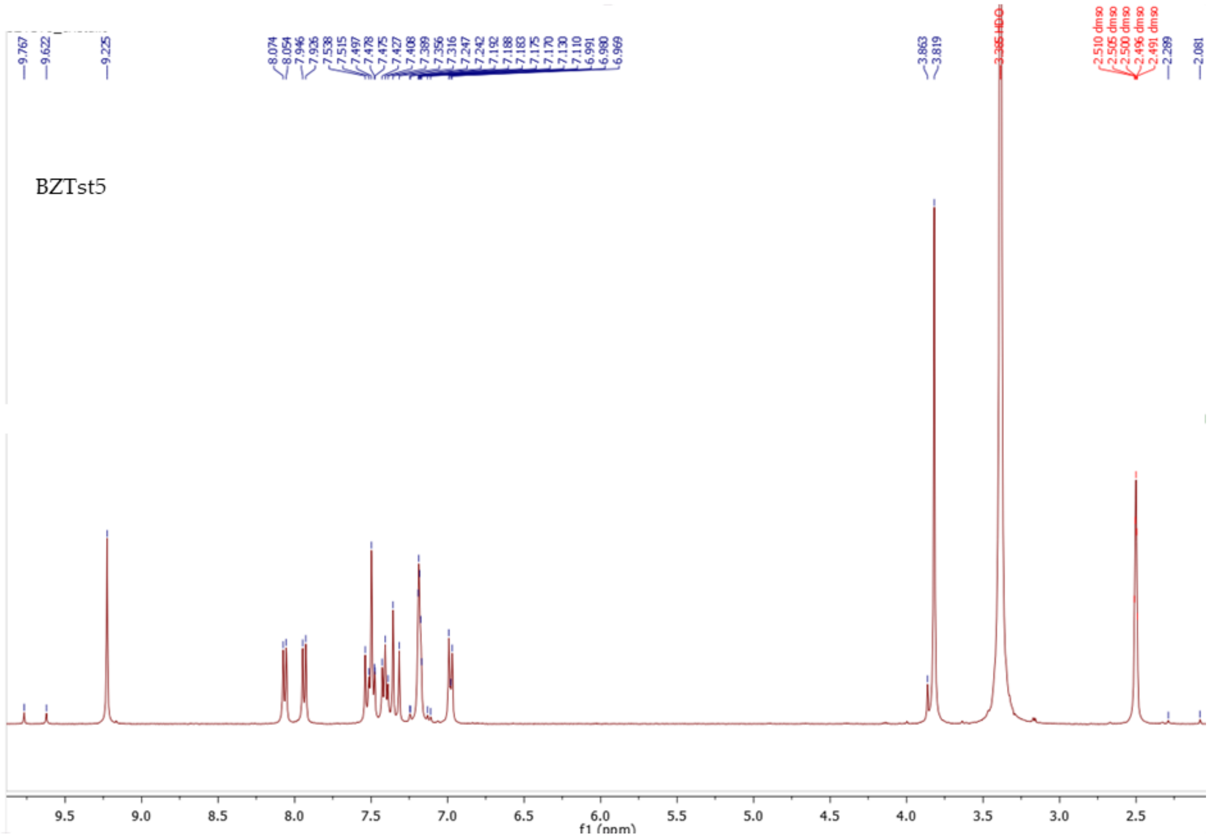

Figure S5. ¹H-NMR spectrum of compound **BZTst5.**

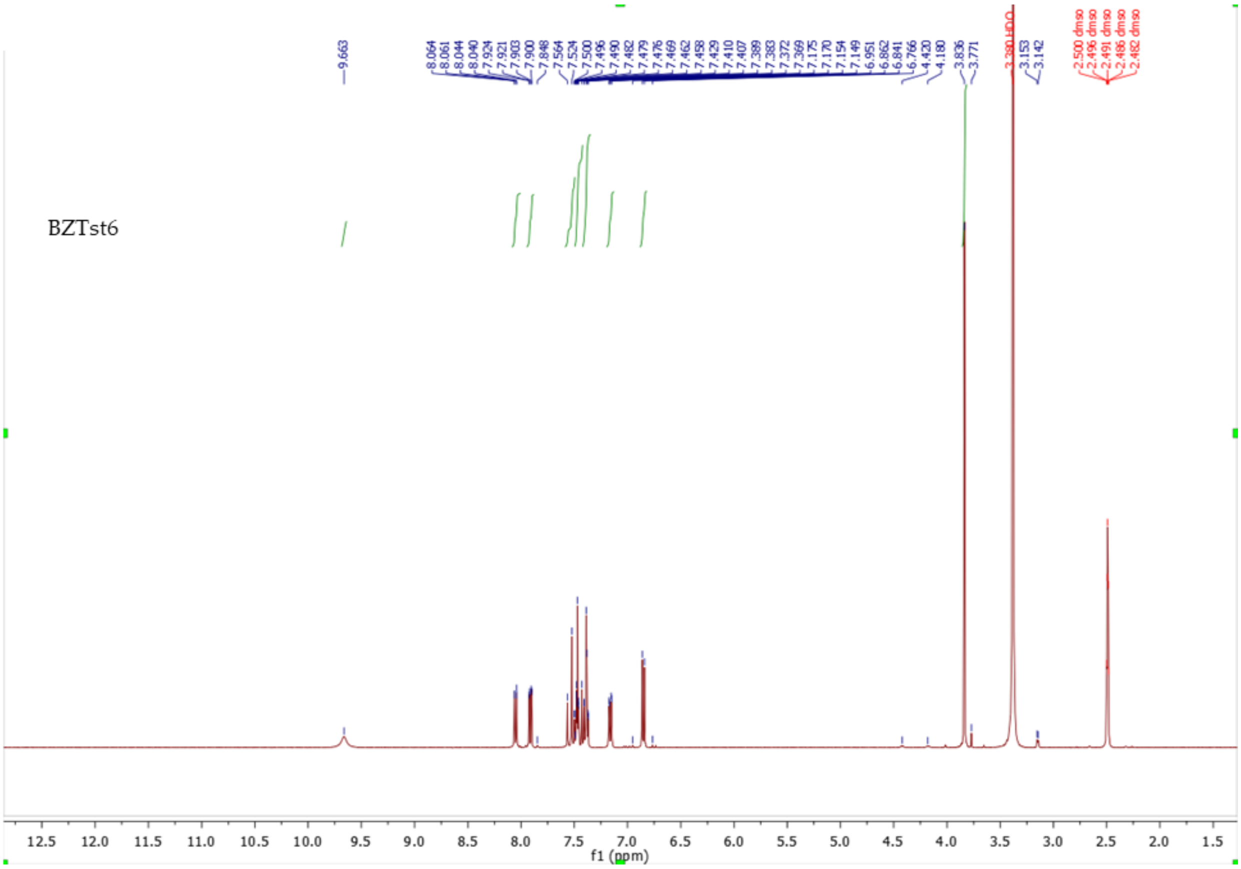

Figure S6. ¹H-NMR spectrum of compound **BZTst6.**

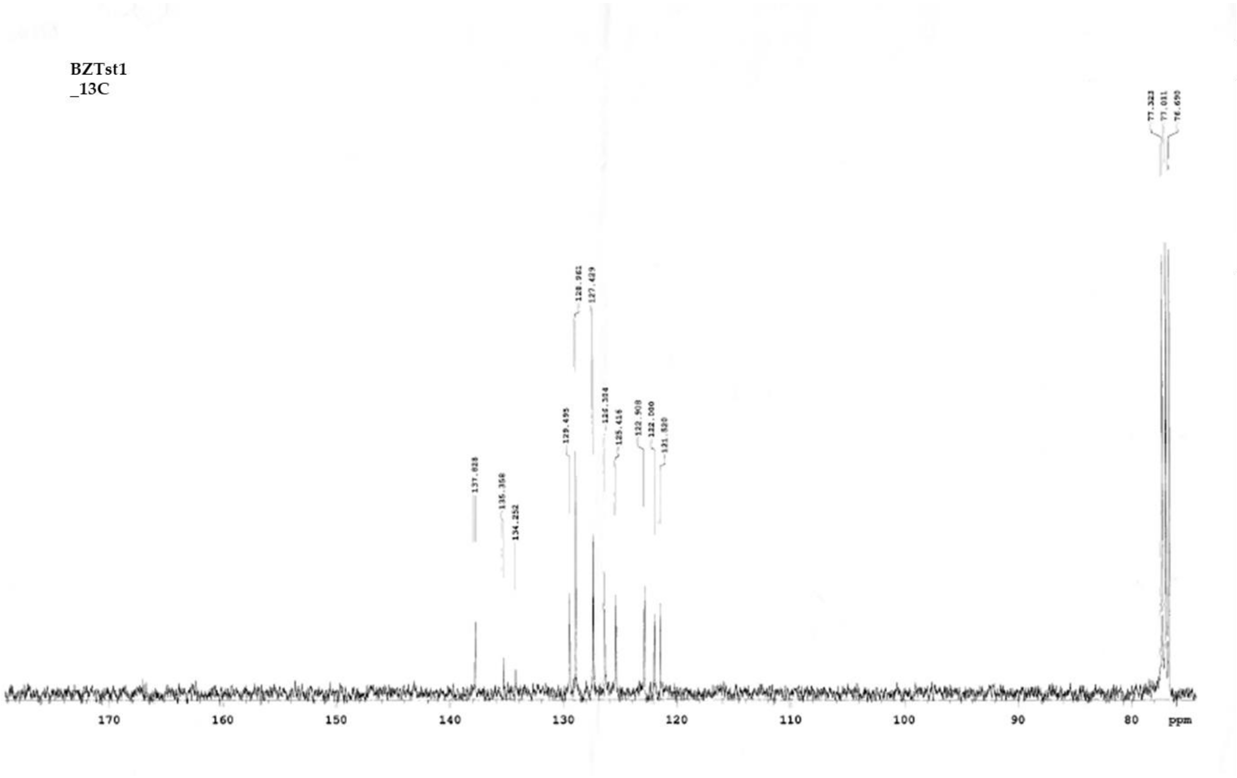

Figure S7. ^{13}C-NMR spectrum of compound **BZTst1.**

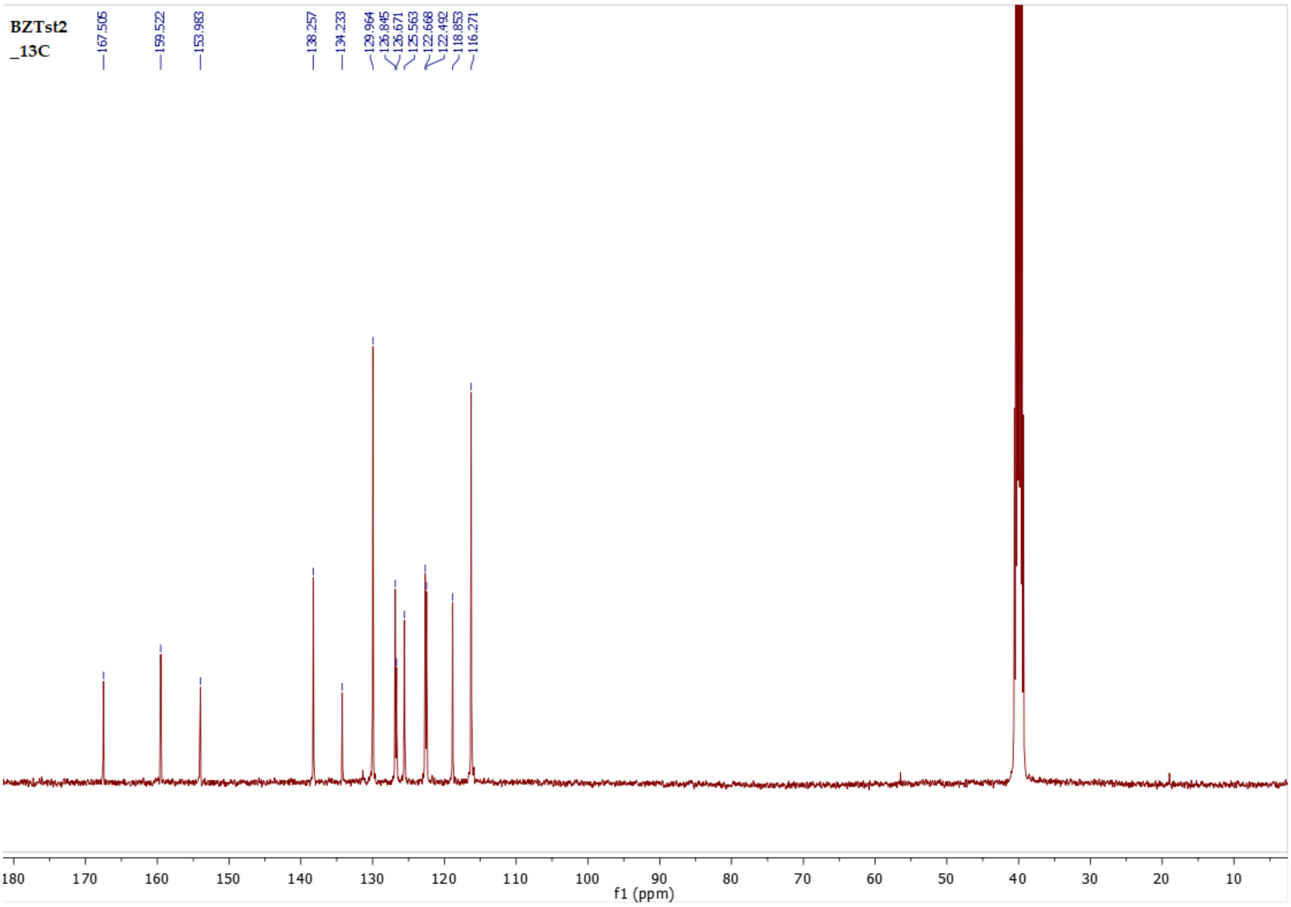

Figure S8. ^{13}C-NMR spectrum of compound **BZTst2.**

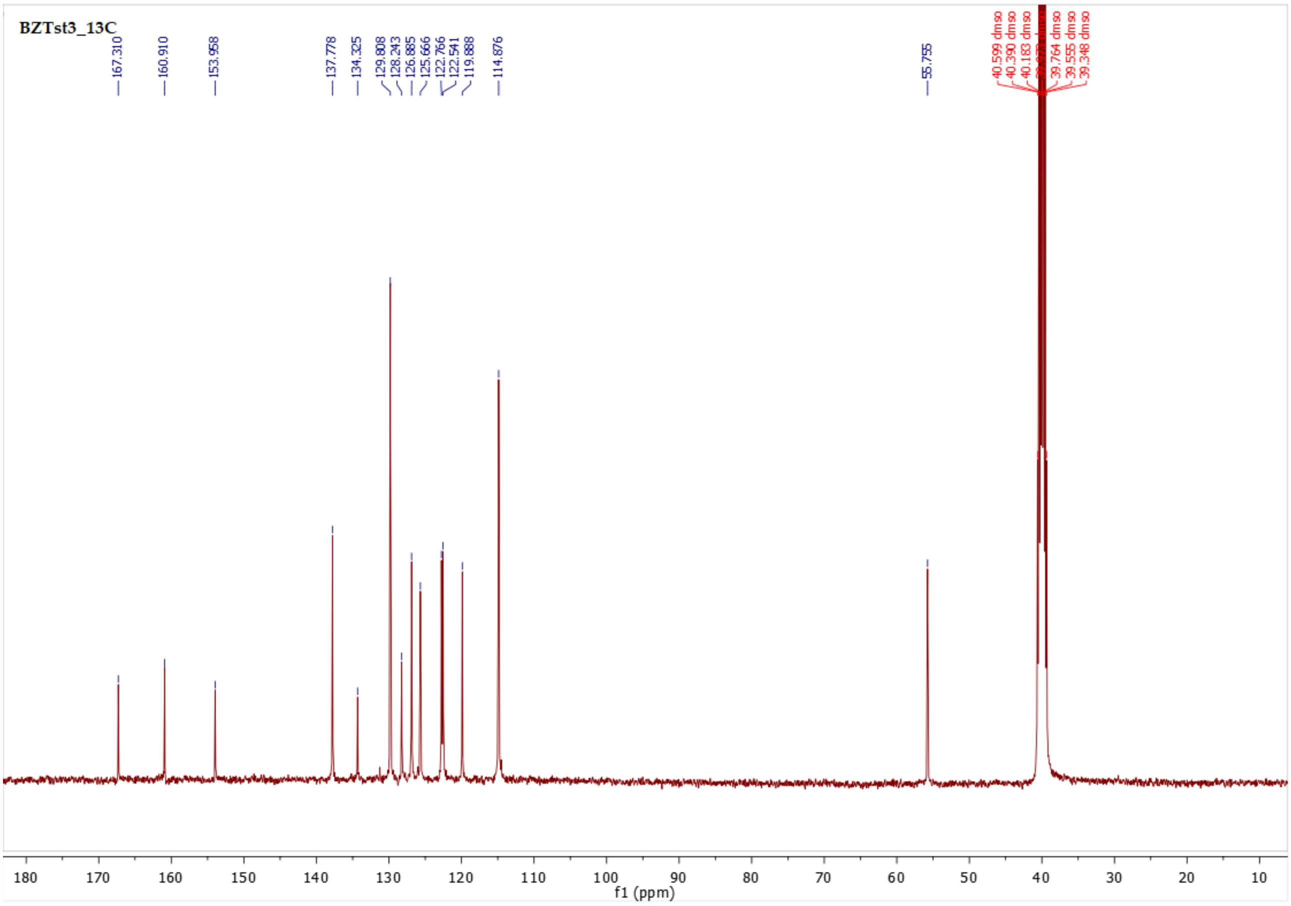

Figure S9. ^{3}C-NMR spectrum of compound **BZTst3.**

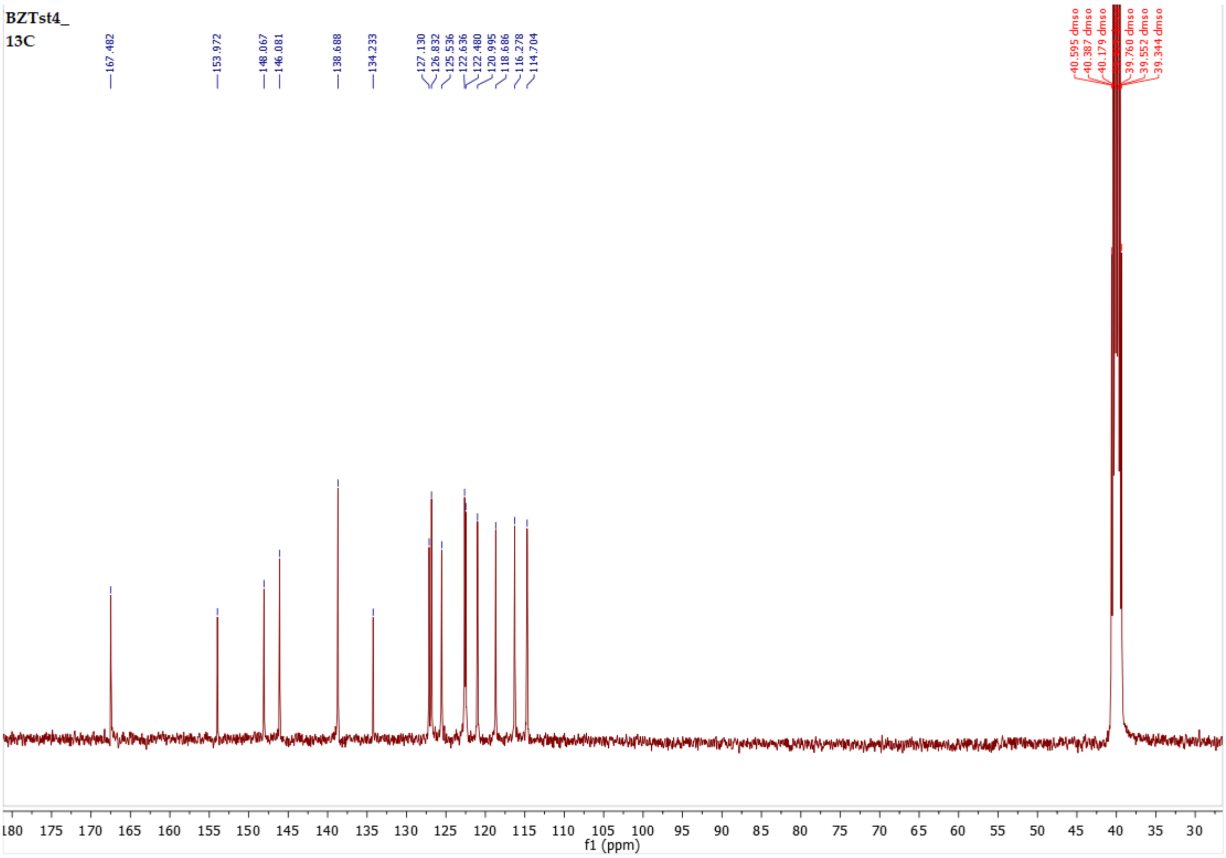

Figure S10. ^{3}C-NMR spectrum of compound **BZTst4.**

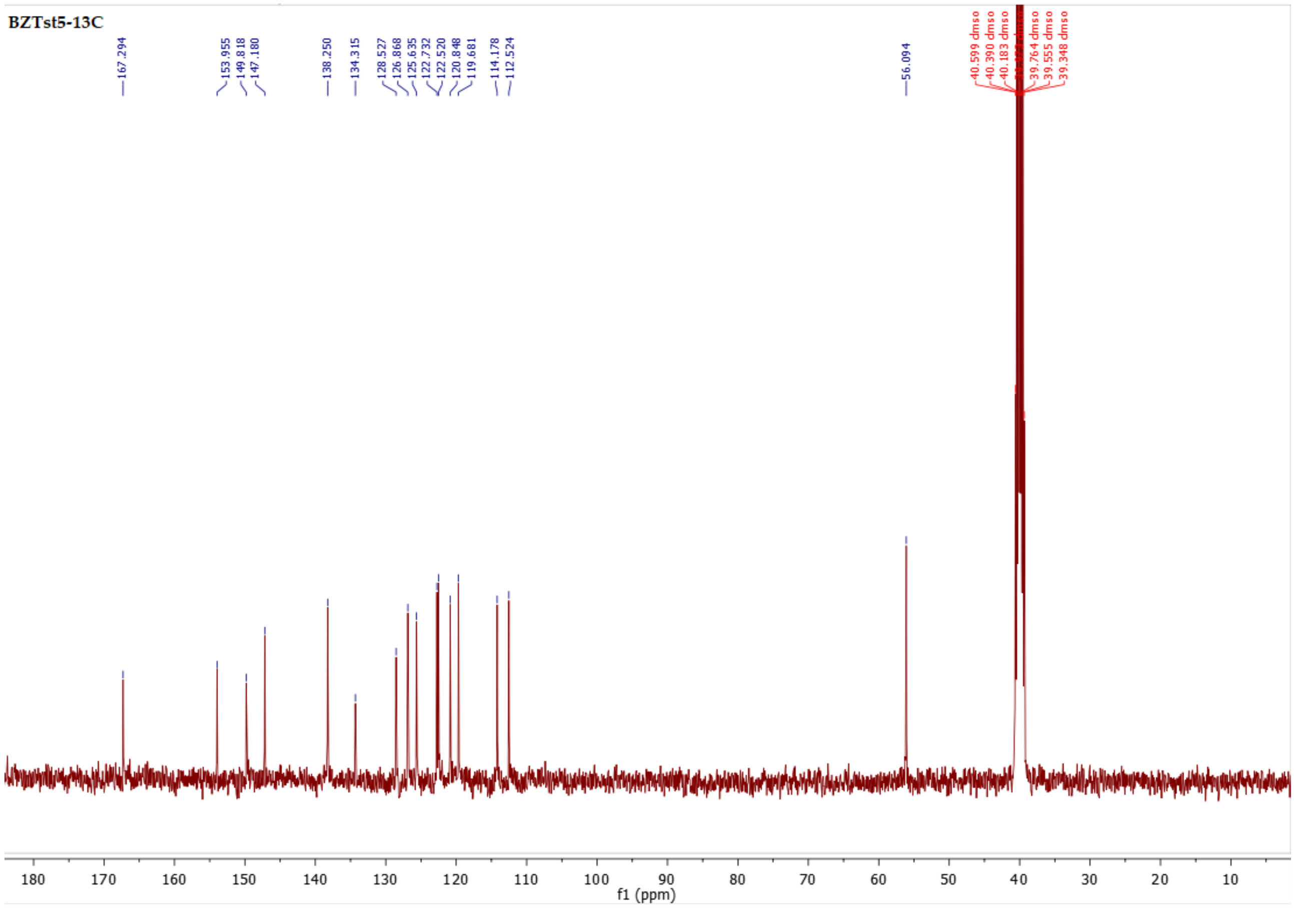

Figure S11. ³C-NMR spectrum of compound **BZTst5.**

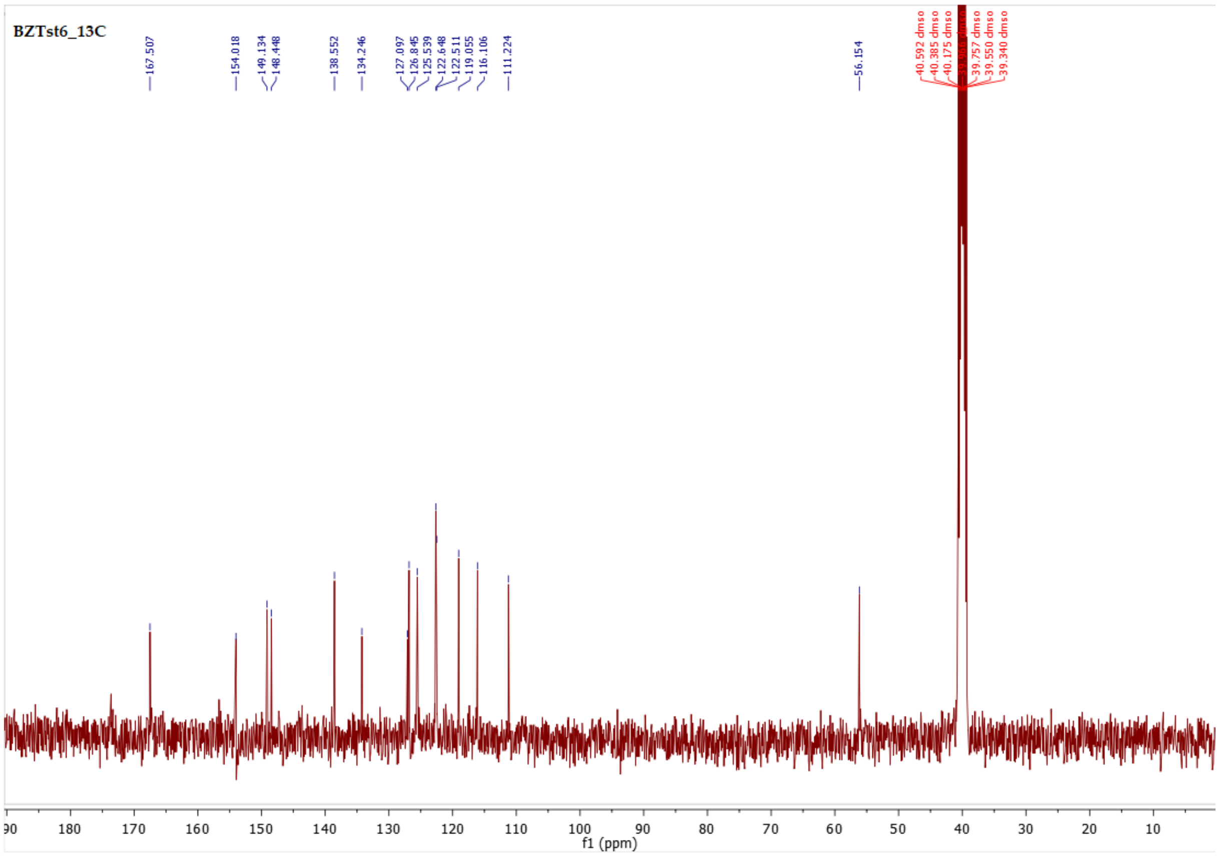

Figure S12. ³C-NMR spectrum of compound **BZTst6**.
